# Strength Analyses of Screws for Femoral Neck Fractures

**DOI:** 10.1007/s40846-018-0378-x

**Published:** 2018-04-20

**Authors:** Karel Frydrýšek, Milan Šír, Leopold Pleva

**Affiliations:** 1 Department of Applied Mechanics, Faculty of Mechanical Engineering, VSB – Technical University of Ostrava, 17. listopadu 15/2172, 708 33 Ostrava, Czech Republic; 2 Trauma Centre, University Hospital Ostrava, 17. listopadu 1790, 708 52 Ostrava, Czech Republic

**Keywords:** Biomechanics, Femoral neck fracture, Cancellous screws, Beams on elastic foundation, Strength analyses, Safe factor

## Abstract

This article represents a multidisciplinary approach to biomechanics (engineering + medicine) in the field of “collum femoris” fractures. One possible treatment method for femoral neck fractures, especially for young people, is the application of cancellous (i.e. lag or femoral) screws (with full or cannulated cross-section) made of Ti6Al4V or stainless steel. This paper therefore aims to offer our own numerical model of cancellous screws together with an assessment of them. The new, simple numerical model presented here is derived together with inputs and boundary conditions and is characterized by rapid solution. The model is based on the theory of beams on an elastic foundation and on 2nd order theory (set of three differential 4th order equations, combination of pressure and bending stress-deformation states). It presents the process for calculating displacements, slopes, bending moments, stresses etc. Two examples (i.e. combinations of cancellous screws with full or cannulated cross-section made of stainless steel or Ti6Al4V material) are presented and evaluated (i.e. their displacement, slopes, bending moments, normal forces, shearing forces and stresses). Future developments and other applications are also proposed and mentioned.

## Introduction

**Fig. 1:**
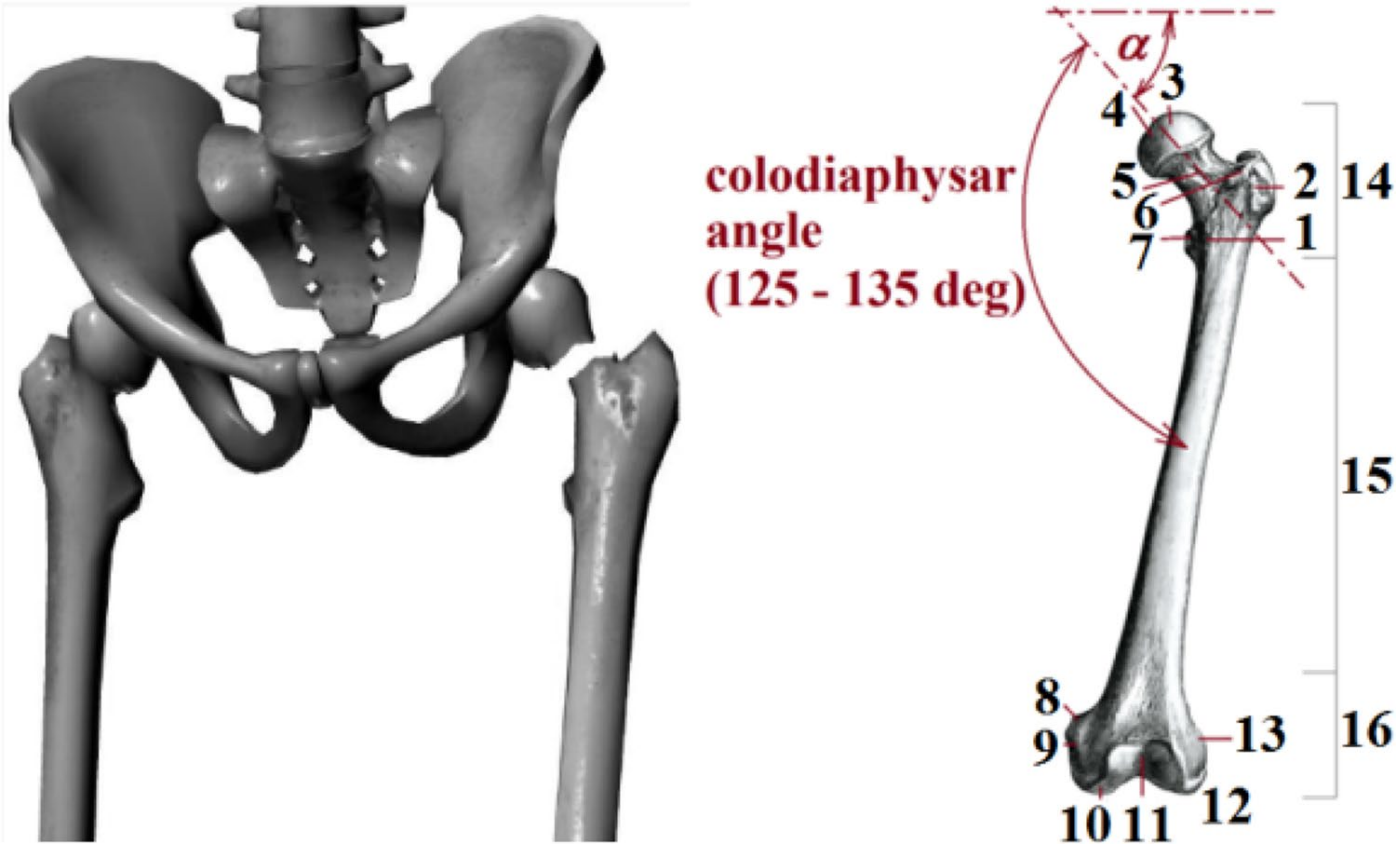
Femoral neck fracture and femur bone (*1*—linia intertrochanterica, *2*—trochanter major, *3*—caput femoris, *4*—fovea capitis femoris, *5*—collum femoris, *6*—tuberositas glutea, *7*—trochanter minor, *8*—tuberculum adductorium, *9*—epicondylus medialis, *10*—condylus medialis, *11*—facies patellaris, *12*—condylus lateralis, *13*—epicondylus lateralis, *14*—proximal end, *15*—diaphysis, *16*—distal end)

Proximal femoral neck fractures (i.e. collum femoris fractures), see Fig. 1, remain a vexing clinical problem in traumatology and orthopaedics and are one of the most common types of trauma, especially amongst elderly patients (women); see [1–14]. As a consequence, femoral fractures are a significant cause of morbidity and mortality in all age groups. One possible treatment method for femoral neck fractures, especially for young people, is the application of cancellous screws (i.e. lag or femoral screws) made from Ti6Al4V or stainless steel materials; see [15].

This article therefore aims to present numerical models (i.e. mostly strength and deformation analyses) of cancellous screws together with a deterministic assessment and a proposal for future experiments and probabilistic reliability assessment (i.e. applications of the Simulation-Based Reliability Assessment (SBRA) Method, Monte Carlo Method etc.); see [3, 4, 16, 17]. The SBRA Method is a modern and innovative approach applied to mechanical structures in engineering.

## Limitations

Although a complex 3D solution was also performed using the finite element method (i.e. CT images were used to create a model of the femur into which cancellous screws were inserted; see [18, 19]), this study focused on a planar model based on a beam resting on an elastic foundation. This planar model is simpler, and above all its solution is quicker, enabling the future generation of random real inputs (loading forces, material properties of screws, length of screws, cross-section and insertion angle of screws, and the stiffness characteristics of the femur substituted by the elastic (Winkler’s) foundation; see [16, 20–23]). For the planar beam model presented here, it is not a problem to conduct millions of random calculations (simulations) in real time using the Monte Carlo method (stochastic simulation of reality).

In our model the cancellous screws are substituted by beams resting on an elastic foundation. Cancellous screws may have various lengths and various positions determined by cancellous screw angle $\propto$ and length ${\text{L}}$, as is the case due to patient anatomy (i.e. our model enables general configurations and numbers of cancellous screws in femur).

In this article, the model only presents the results for cases of screws with full or cannulated cross-section inserted in parallel positions (i.e. the easiest mathematical case). However, the other (i.e. general) positions of cancellous screws in femur can be solved too. Changes of angles $\propto$ and length ${\text{L}}$ can be reflected by simply changing the screw positions in the model, thus enabling us to evaluate appropriate, less appropriate or inappropriate cancellous screw positions for operations.

This article focuses primarily on biomechanics (methodology for determining forces, stress and deformations in cancellous screws); it does not attempt to assess and evaluate traumatological/orthopaedic treatment methods.

The nature and simplicity of the elastic foundation used in this article makes it an attractive and significant simplification of the generally very complex interactions between screws/implants and bones or other human tissues. The choice of stiffness for the elastic foundation is directly influenced by the material properties of bone, which vary depending on each individual patient and are thus generally random (stochastic).

This is not a solution of a direct dynamic problem. Nevertheless, the influence of dynamic effects is reflected in the dynamic coefficient which increases the static force based on the mass of the patient; this is a generally accepted engineering approach.

The material of the cancellous screws is linear, isotropic and homogeneous.

The material properties of the femur, and thus the interaction between the cancellous screw and the femur, are substituted by the elastic foundation.

In philosophy, it is our opinion that “strength lies in simplicity”, and for this reason we have developed a planar and linear model (i.e. the generally complex spatial problem of positioning cancellous screws in the femur is simplified).

From the perspective of orthopaedics/traumatology there is a relatively large quantity of information and statistical evaluations of treatment methods. Nevertheless, from a biomechanical perspective there is an absence of descriptions of numerical models which would enable us to evaluate the appropriateness of screw positions or the selection of operating techniques from an engineering/biomechanical point of view (mechanical stress, deformation of screws or bone). *The article does not directly evaluate any specific operating technique; it merely presents a new, original model including its mathematical/biomechanical basis and basic results.*

## Materials and Methods

Beams on elastic foundations are frequently used in mechanical, civil, mining, marine, soil, geotechnical and other types of engineering.

The elastic foundation (linear/nonlinear) can also be applied if a physical object (such as an implant or bone) is supported/embedded; see [16, 24, 25]. In general (engineering point of view), the mechanical behaviour of periosteum, compact and spongy bone or even soft and porous tissues can be approximated via elastic foundations with an appropriate definition of stiffnesses; see [4, 16, 18, 23, 25–30]; hence, the elastic foundation is a suitable approximation/simplification for mechanical contacts. Therefore, from the biomechanical perspective, the cancellous screw is described and solved here as a beam on an elastic foundation.

The numerical model is derived from and based on the theory of beams on an elastic (Winkler’s) foundation (i.e. a set of three differential 4th order equations with twelve boundary conditions, combined pressure and bending stress-deformation states), where the bone is approximated by the elastic foundation. Hence, the cancellous screw is resting along its whole length ${\text{L}}$ on an elastic foundation prescribed by stiffness $k$ (i.e. the elastic foundation surrounds the whole screw); see [16, 18, 23–25].

The value of stiffness $k$ depends on the mechanical properties of the femur. For example, if the cancellous screw is in contact with the cortical bone (generally accepted medical practice), stiffness $k$ must be greater than if the screw is not in contact. In our case, the correct choice of stiffness $k$ enables us to describe the general position of the screw in the proximal part of the femur.

Three screws of length ${\text{L}}$ were applied in parallel positions on the elastic foundation (i.e. in the femur) and were loaded by total quasi-dynamical force ${\text{F}}_{\text{m}}$ acting on the direction of the cancellous screw angle ∝; see Fig. 1.

For medical professionals it is important to emphasize the following points. The computational model presented here can also be applied for situations when the number of cancellous screws is lower or higher than 3. Also, angle ∝ can be different for each cancellous screw (common medical practice); however, in view of the anatomy of the proximal femur, in such cases there is also a change in the length of the individual cancellous screws, as well as in the distribution of forces; our computational model also respects this fact. It is our opinion that “strength lies in simplicity”, and for this reason we have developed a planar and linear model (i.e. the generally complex spatial problem of positioning cancellous screws in the femur is simplified).

From a biomechanical perspective, our model can be used to perform a relatively simple assessment of the general position of cancellous screws in the femur (i.e. it can assess appropriate, less appropriate and inappropriate screw positions for purposes of osteosynthesis following collum femoris fractures). However, it is not our primary goal in this paper to assess or propose medical techniques.

Typical shapes and dimensions of the cancellous screws are presented in Table 1 (screws made by MEDIN a.s., Nové Město na Moravě, Czech Republic; see [19]).

**Table 1:**
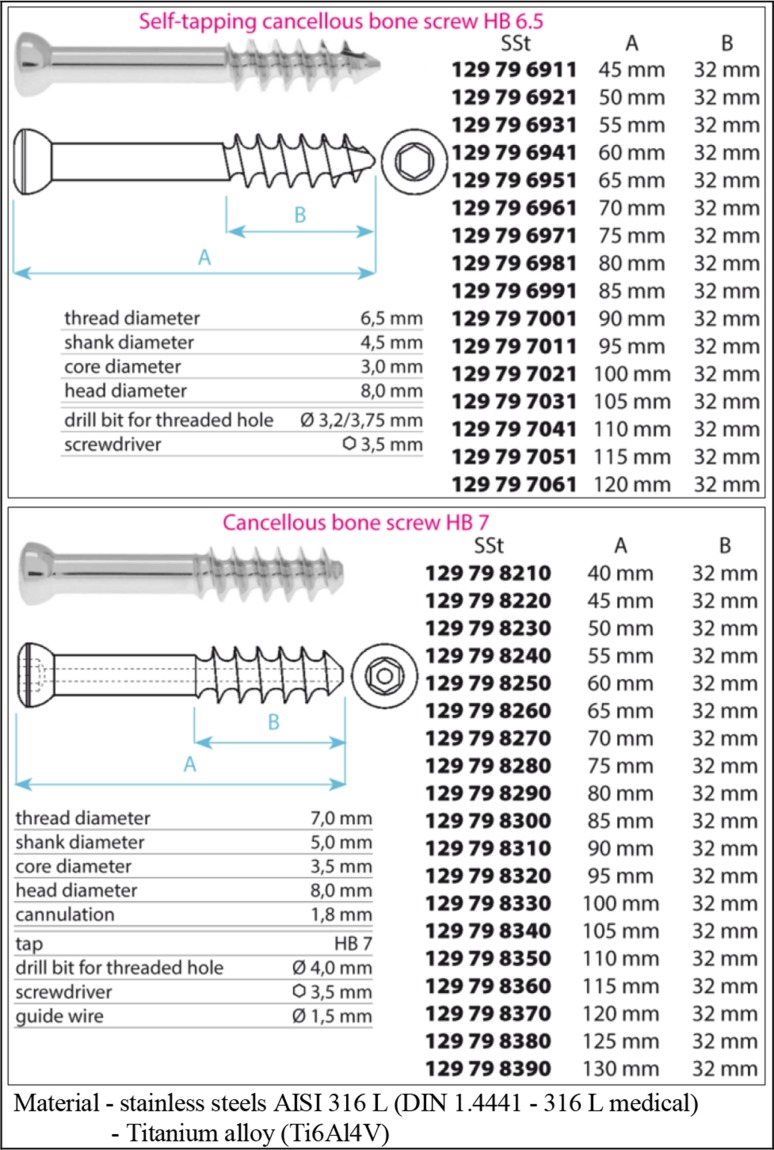
Cancellous screws (producer MEDIN a.s.; see [19])

The stainless (corrosion-resistant) steels (for example AISI 316 L, DIN 1.4441—316 L medical) used nowadays to produce implants are primarily high-alloy austenitic steels with high Cr, Ni and Mo content and low carbon content. This chemical composition gives good resistance against most types of corrosion, including intercrystalline and point corrosion. However, it is not resistant to fretting corrosion.

Titanium and its alloys (for example Ti6Al4V, see [31, 32]) usually have excellent properties and inertness. They give a high degree of corrosion resistance—both when exposed to air and in the chemically aggressive environment of the human body. They also retain their positive properties at low and high temperatures.

## Medical Perspective

The anatomical area of the proximal femur, see Fig. 1, consists of the femoral head (caput femoris) and neck (collum femoris), together with the trochanteric area, trochanter major and trochanter minor.

**Fig. 2:**
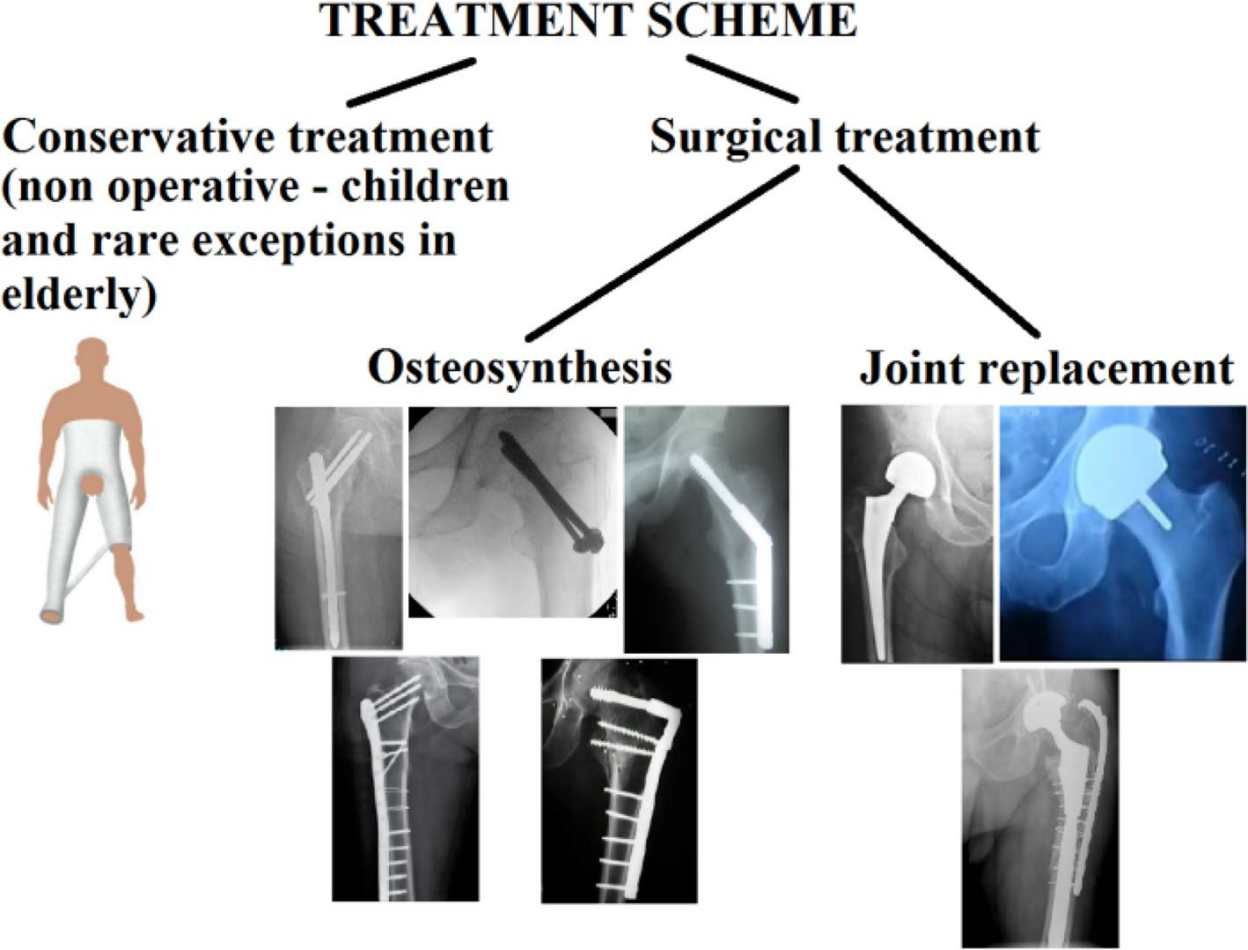
Treatment of proximal femoral fractures

Proximal femoral fractures, see Figs. 1a and 2, are one of the most commonly observed fractures. Annually, approximately around 10,000 to 15,000 of these accidents occur in the Czech Republic; see [1]. The number reaches up to 900,000 cases in Europe every year. For more information see [1, 6, 11–14].

The treatment of proximal femoral fractures, see Fig. 2, is associated, apart from therapeutic problems, also with social and economic issues, taking into consideration the long period of treatment. In young patients, this type of fracture occurs especially due to high-energy mechanisms, such as traffic accidents, falls from height, and also adrenaline sports. In older individuals, the fractures are most frequently caused by low-energy injuries, e.g. falls at home. The first (and less frequently observed) group of fractures comprises fractures of the femoral head, which most frequently occur during dislocation of the hip joint.

Femoral neck fractures may be divided into intracapsular fractures (i.e. fractures in the hip joint space) and extracapsular fractures (i.e. fractures located outside the articular capsule). Intracapsular fractures may be further divided into subcapital and mediocervical fractures. In the case of extracapsular fractures, we can differentiate between basicervical and trochanteric fractures. From the perspective of healing, extracapsular fractures are associated with a better prognosis, because in intracapsular fractures the vascularization in the fracture area is usually also disrupted, which is associated with healing disorders.

**Fig. 3:**
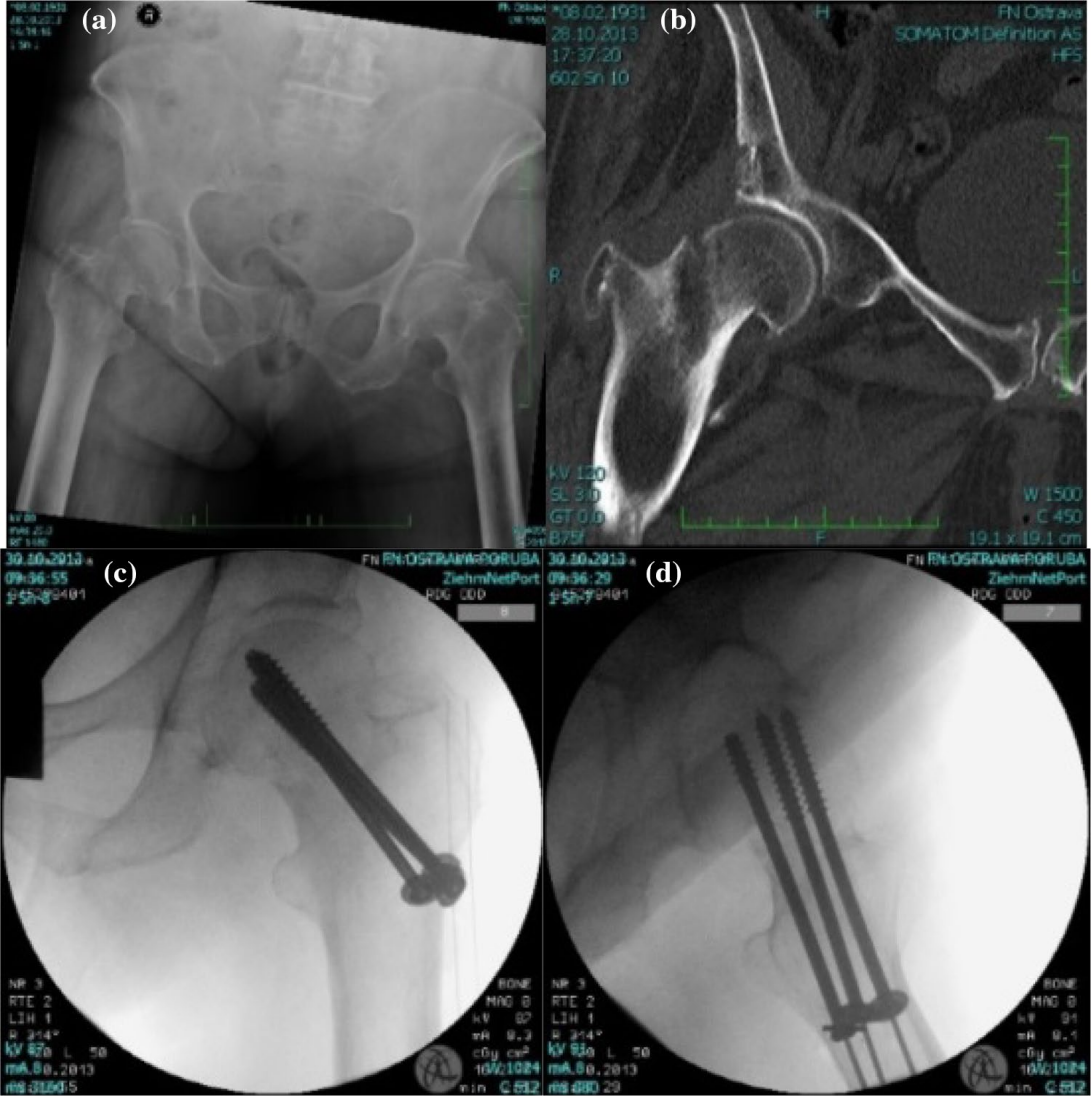
Femoral intracapsular fracture **a** pelvic X-ray, **b** CT scan, **c** Osteosynthesis with three lag spongious screws, **d** Osteosynthesis with three lag spongious screws

The type of osteosynthesis in intracapsular fractures depends mainly on the age of the patients. In young individuals, procedures which preserve the femoral head are usually chosen; this is based on the assumption that a few patients may require a joint replacement at a later stage, following the development of avascular necrosis (AVN). There is a choice between osteosynthesis performed with lag cancellous screws and the DHS procedure, with placement of antirotation screws. Identical methods are also suitable for nondislocated and minimally dislocated fractures in patients of higher age; see Fig. 3.

**Fig. 4:**
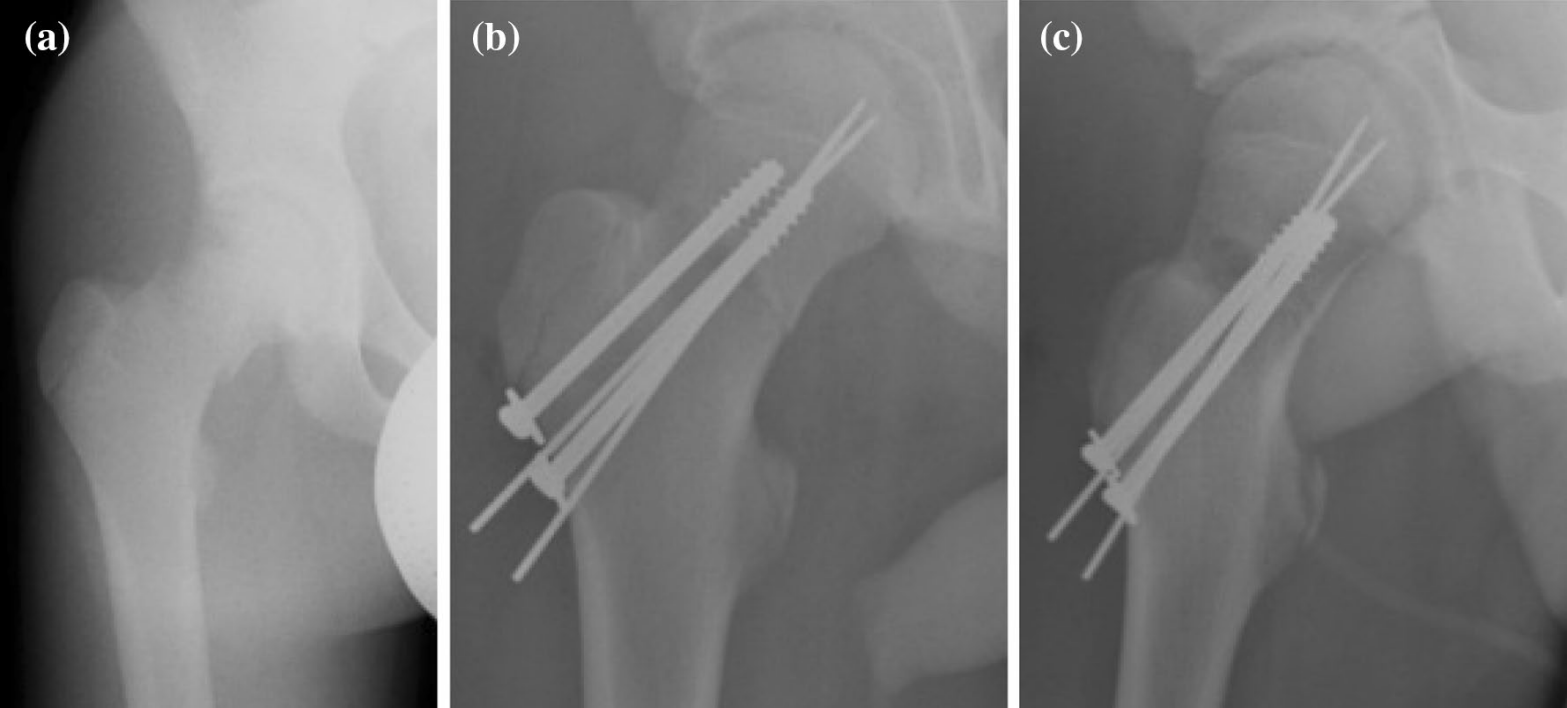
Child’s intracapsular femoral fracture **a** anteroposterior X-ray, **b** Osteosynthesis with two lag spongious screws and two Kirschner wires through the epiphyseal zone (anteroposterior X-ray), **c** Osteosynthesis with two lag spongious screws and two Kirschner wires through the epiphyseal zone (lateral X-ray)

Osteosynthesis with lag spongious screws is also clearly indicated in cases of proximal femur fractures in children, in combination with osteosynthesis using Kirschner wires introduced through the epiphyseal growth zone; see Fig. 4.

Replacements of the hip joint are indicated especially in older patients with dislocated intracapsular fractures, in the presence of advanced coxarthrosis of the affected joint.

At the Trauma Centre of the University Hospital in Ostrava (Ostrava, Czech Republic), approximately 300 patients undergo surgery annually due to proximal femur fractures.

**Fig. 5:**
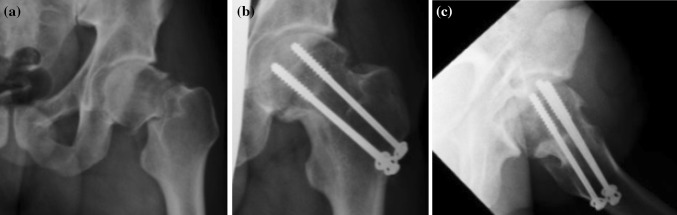
Intracapsular femoral fracture **a** anteroposterior X-ray, **b** Osteosynthesis with three lag screws (anteroposterior X-ray), **c** Osteosynthesis with three lag screws (lateral X-ray)

**Fig. 6:**
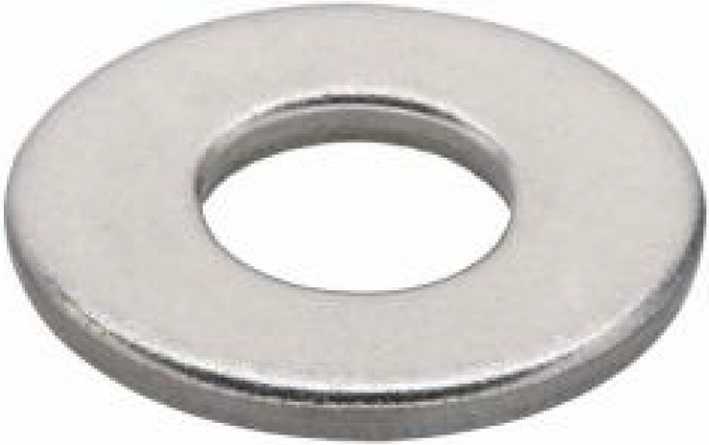
Underlay for cancellous screw

Osteosynthesis of femoral neck fractures with lag spongious screws is usually performed on children and young patients with intracapsular fractures, but also on patients of a higher age with nondislocated intracapsular fractures; see Fig. 5. The use of lag spongious screws belongs among the mini-invasive techniques; the placement of two or more screws provides rotational stability. Length stability is provided by the placement of the screw tips into the subchondral bone and by supporting the screw head with an underlay, see Fig. 6.

Hence, the treatment of collum femoris fractures is one of the most common procedures solved and performed by orthopaedists/traumatologist; see Table 2. This article therefore focuses on their biomechanical modelling (i.e. primarily strength/deformation analyses and their evaluation via safety factor of cancellous screws).

**Table 2:** Treatment of collum femoris fracture

Femoral neck fracture screws	
Indications	Nondisplaced femoral neck fracture. Displaced femoral neck fracture in young and active patients
Contraindications	Displaced femoral neck fracture in elderly, inactive patients. Rheumatoid arthritis, moderate osteoarthritis, poor bone density, limited life expectancy and pathologic fracture
Alternatives	Hemiarthroplasty, total hip arthroplasty (THA) and dynamic hip screw with derototation screws

Note that the medical perspective (i.e. orthopaedics/traumatology in this chapter) is not the main focus of our work. The goal is to present the model from a biomechanical perspective, as described in the following chapters.

## Osteosynthesis Via Three Cancellous Screws (Beams)

**Fig. 7:**
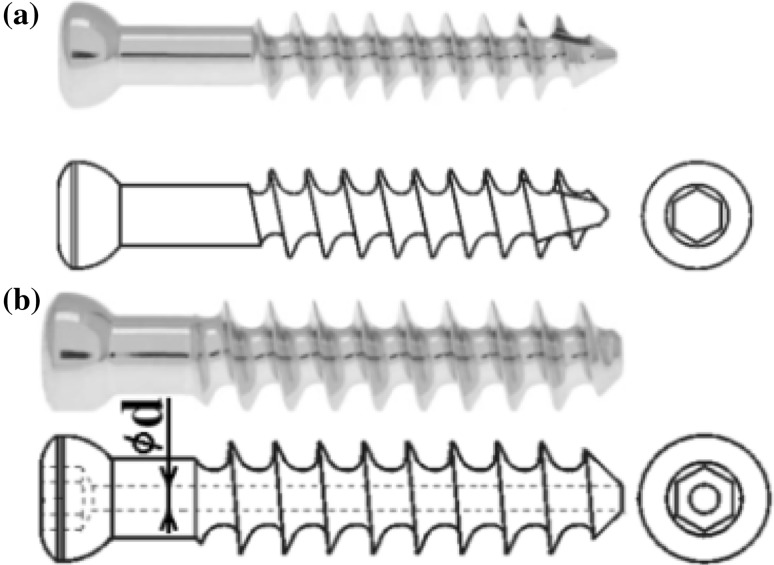
Cancellous screws **a** with full cross-section **b** with cannulated cross-section (producer MEDIN a.s.; see [19])

Cancellous screws (i.e. lag spongious screws or femoral screws as mentioned previously; see Table 1) can be produced with full or cannulated cross-section, see Fig. 7. They are usually made of medical stainless steel (AISI 316 L, DIN 1.4441—316 L medical) or Ti6Al4V material; see Chap. 2. Materials are a key factor in ensuring the full functionality of each implant.

This study uses screws made by MEDIN a.s. (Nové Město na Moravě, Czech Republic, see [19]), though the methods and results can also be applied for other types of screws. The aim is to perform strength and deformation analyses of cancellous screws and to evaluate the results.

**Fig. 8:**
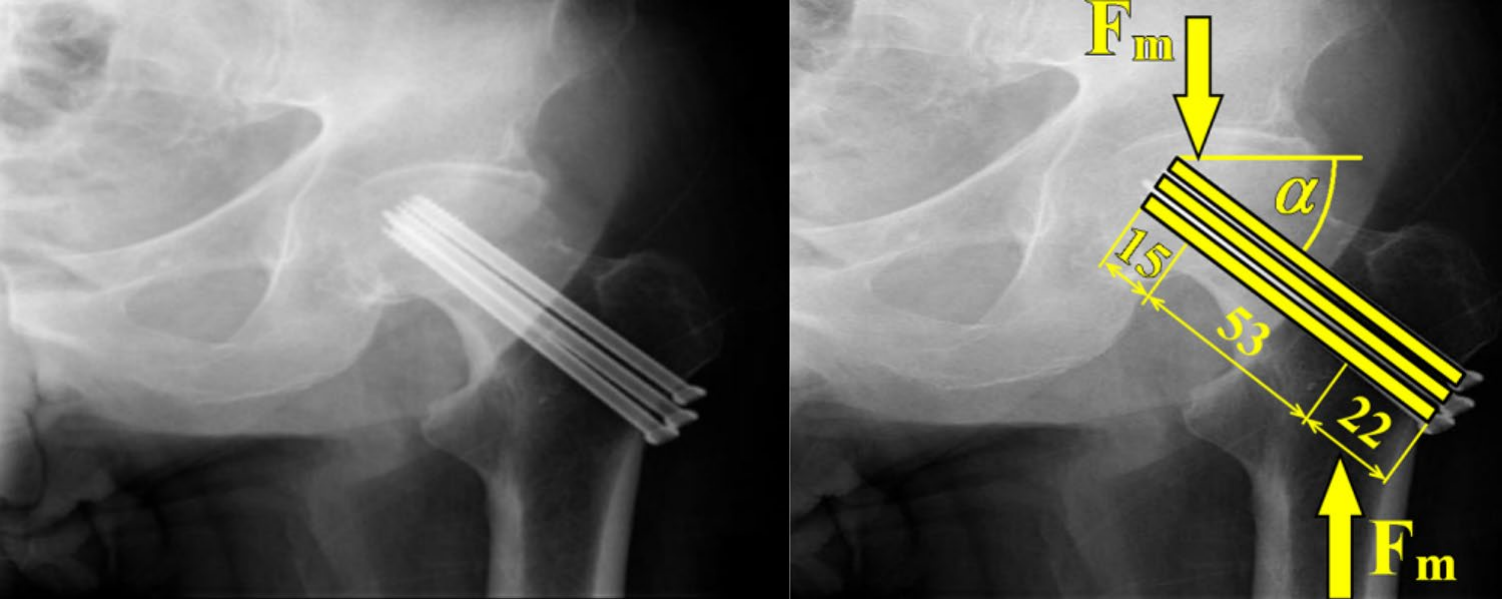
Three cancellous screws (X-ray image) and their approximation via parallel beams on an elastic foundation

**Fig. 9:**
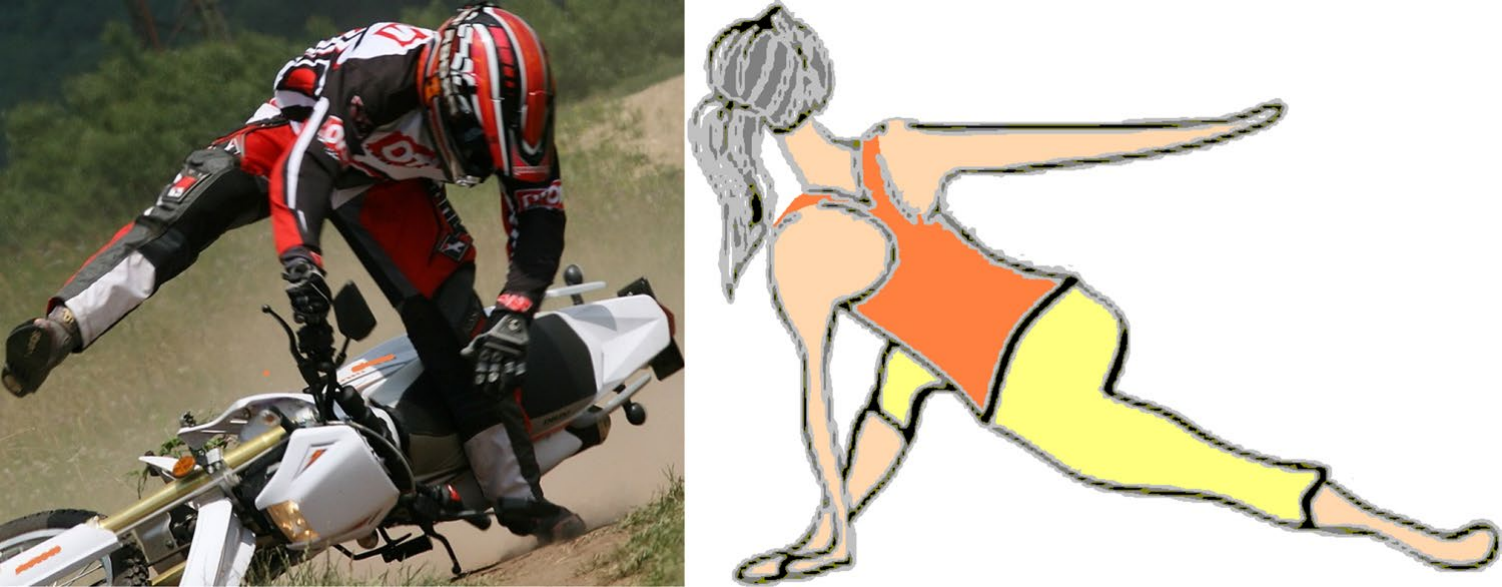
Main definition for limiting values of cancellous screw angle $\propto$

Hence, the aim is the biomechanical solution of osteosynthesis via three cancellous screws loaded by total quasi-dynamical force ${\text{F}}_{\text{m}}$ acting on the direction of cancellous screw angle $\propto$; see Figs. 1 and 8 and references [12, 18, 33, 34]. Force ${\text{F}}_{\text{m}}$ is evoked by the movement of the human body. The cancellous screw angle $\propto$ (which is connected with force ${\text{F}}_{\text{m}}$ and which is defined primarily by the limiting angles of adduction and abduction and secondarily by the screw insertion angle in osteosynthesis) lies between 5 and 80 deg. A typical value for angle $\propto$ is 50 deg. However, the real variability of angle $\propto$ is taken into account by the probabilistic approach (possible future development, i.e. $\propto \in (5; 80)$  deg; see Fig. 9 and Ref. [18]).

Whole parts of screws can be considered as beams on elastic foundations, and in the bone they are in approximately parallel positions; see Fig. 9. However, in general, the screws do not need to be in parallel positions (i.e. our computational model also takes this possibility into account).

Note that in references [20, 21] (i.e. the relevant medical point of view) the cancellous screws (i.e. beams) are in different configuration (i.e. Biplane Double-Supported Screw Fixation Method) and are considered as beams with overhanging ends, and usually without elastic foundations (the simplest numerical model).

Applications of elastic foundations (i.e. Winkler’s foundations) offer a simple but fast and acceptable solution of the problem. Hence, the elastic foundation is a suitable approximation for the femur body. For more information about elastic foundations see references [16, 23–25].

## Loading of Cancellous Screws (Beams)

**Fig. 10:**
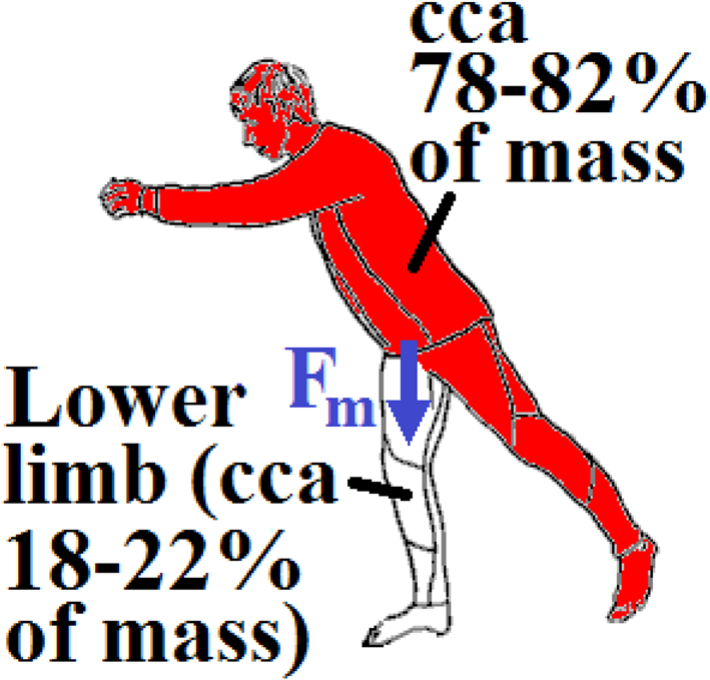
Loading of cancellous screws—a man standing on one leg

The patient of total mass ${\text{m}}$ is standing on one leg (i.e. maximal loading acting on the femur); see Fig. 10. The mass of a lower limb is 18–22% of the entire body (i.e. the mass of the body without one lower limb is 78–82% of the entire body); see [18, 34]. This fact is taken into consideration by the coefficient ${\text{k}}_{\text{m}} \in (0.78;\,0.82)$.

**Fig. 11:**
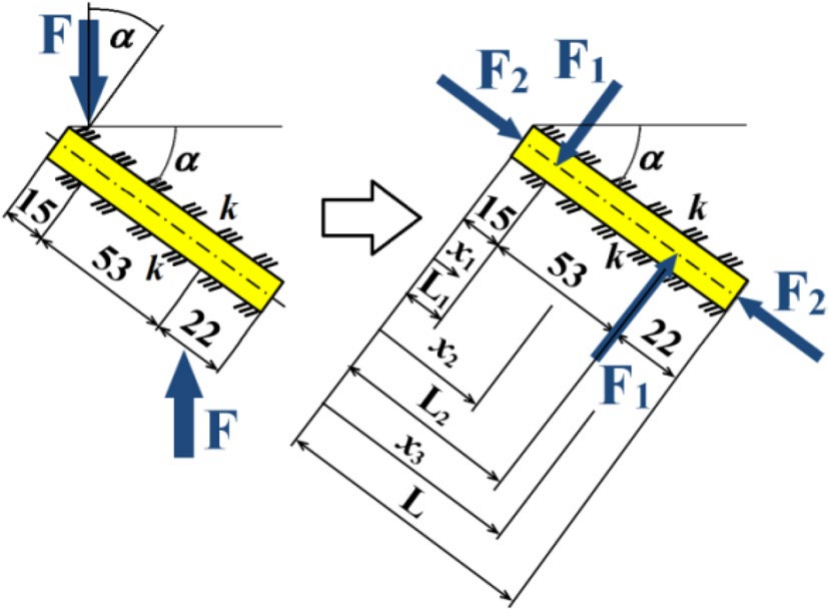
Loading of one cancellous screw and coordinate system

Total loading quasi-dynamic force ${\text{F}}_{\text{m}}$ acting in the caput femoris can be written as

1 $${\text{F}}_{\rm m} = {\text{m}} \times {\text{k}}_{\rm m} \times {\text{k}}_{\rm dyn} \times {\text{g,}}$$

where ${\text{k}}_{\text{dyn}} \in (1; 4)$ is the dynamic force coefficient (taking into account additional dynamic effects such as jumps, falls etc.) and ${\text{g }} = 9.807$  m/s^2^ is gravity acceleration. Upper force ${\text{F}}_{\text{m}}$ is acting in the centre of the caput femoris and lower force ${\text{F}}_{\text{m}}$ is acting in the femoral shaft axis; see Figs. 8 and 10. Force ${\text{F}}_{\text{m}}$ is divided into three screws (beams). Hence in one beam force ${\text{F}}$, see Fig. 11, is defined via the expressions

2 $${\text{F}} = {\text{F}}_{\text{m}} /{\text{n,  }}\,{\text{F}}_{1} = {\text{F}} \times \cos \left( \propto \right),\,   {\text{F}}_{2} = {\text{F}} \times \sin \left( \propto \right),$$

where ${\text{n}}$ is the coefficient of inequality in the division of forces, ${\text{F}}_{1}$ is tangential force and ${\text{F}}_{2}$ is axial force; see Fig. 11.

Coefficient ${\text{n}}$ respects possible variations of maximal and minimal values of force ${\text{F}}_{\text{m}}$. There are two limits. If ${\text{n}} = 3$, then force ${\text{F}}_{\text{m}}$ is uniformly distributed on all beams (minimal value, i.e. divided by 3), and if ${\text{n}} = 2$, then force ${\text{F}}_{\text{m}}$ is nonuniformly distributed and acting only in two beams (maximal value, unfavourable state). However, the reality of this can be taken into account by probabilistic inputs (possible future development, i.e. ${\text{n}} \in (2; 3)$; see [18].

Our numerical model presupposes that there is a primary axial pressure in the beam and no relative movement between both parts of a broken collum femoris. This is performed by axial forces ${\text{F}}_{2}$; see Fig. 11.

The real interference between the femur and screws (beams) can be approximated according to the theory of beams on an elastic foundation by stiffness $k$; see [16]. Thus, bone tissue surrounded the screw in a similar way as an elastic foundation surrounded the beam.

**Fig. 12:**
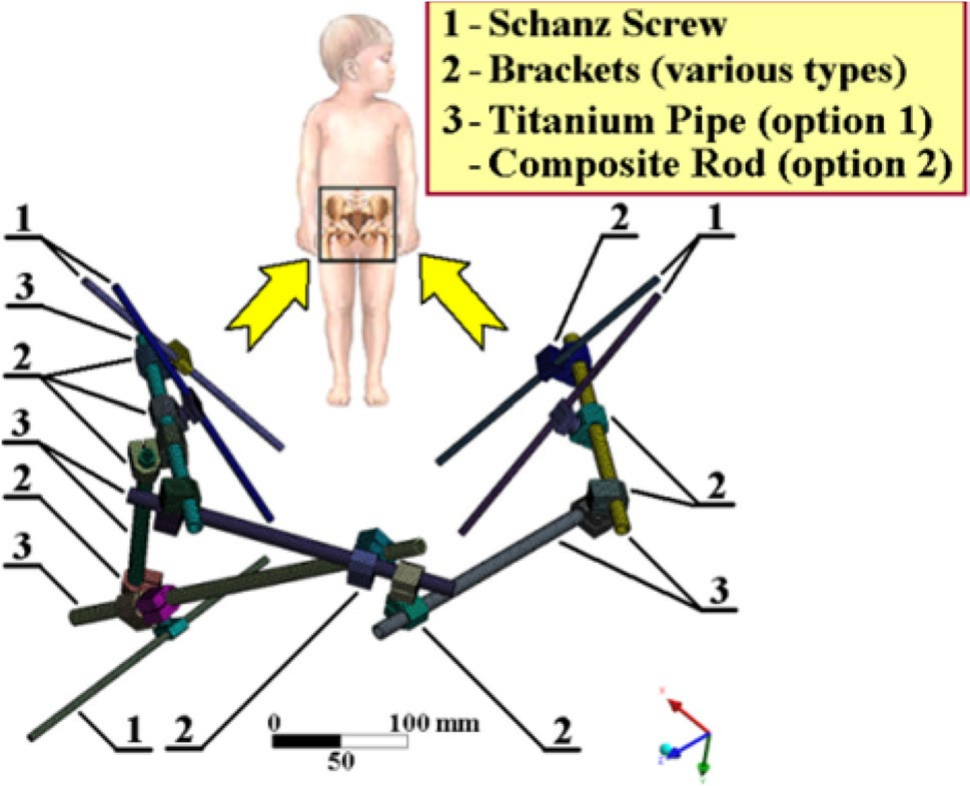
Another application of an elastic foundation in biomechanics (external fixator for treatment of combined pelvic and acetabular fractures); see [4]

There has been extensive experience with approximations of bones via an elastic foundation; see Fig. 12 (i.e. the solution of an external fixator for the treatment of combined pelvic and acetabular fractures, where the interaction between Schanz screws and the pelvis and its acetabulum is described via an elastic foundation) and e.g. references [4, 16, 20, 21, 26].

The typical diameter of the cancellous screw is the shank diameter ${\text{D}}$, which is used in the following solution. Note that if the bone has grown well around the screw (i.e. the normal situation after several weeks of complication-free treatment), the influence of the notch effect of the screw thread shape on mechanical stress and deformation (deflections and slopes) in the screw is small. The characteristic diameter of the screw (beam) can be considered as the screw shank diameter D, which approximately corresponds with the mean diameter of the threaded part.

However, the cannulated cancellous screws also have their inner diameter ${\text{d}}$; see Fig. 7 and Table 1.

References [20, 21] (i.e. the medical perspective) are also focused on cancellous screws solved as beams on elastic foundations. However, the solution in these references is different, being performed for one loading force ${\text{F}}$, while the influence of axial forces is neglected and the elastic foundation is only mentioned in passing. In our opinion, this is the simplest approach ([20, 21]) but it is not sufficiently accurate.

## Cancellous Screws as Beams on an Elastic Foundation

**Fig. 13:**
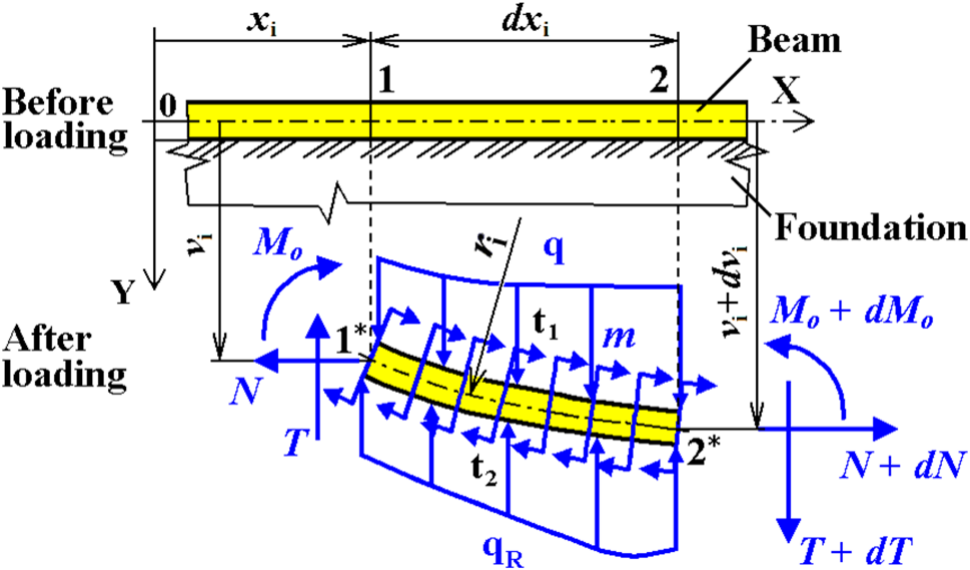
Element of a beam on elastic foundation (general formulation; see [16])

In mechanics/biomechanics, the analysis of bending of beams on an elastic foundation is developed on the assumption that the strains are small and the distributed reaction forces ${\text{q}}_{\text{R}}$ in the foundation are proportional at every point to the deflection $v_{\text{i}}$ of the beam at that point etc.; see Fig. 13.

In the most situations, the influences of temperature ${\text{t}}_{1}$ and ${\text{t}}_{2}$, distributed moment $m$ and distributed loading q can be neglected (or the beam is not exposed to them).

According to [16] and Fig. 13 (i.e. 2nd order beam theory—direct influence of tensile/compression and bending loading), the general formulas for beams on an elastic (Winkler’s) foundation (i.e. the solution of a 4th order linear differential equation)

3 $$EJ_{\text{ZT}} \frac{{d^{4} v_{\text{i}} }}{{dx_{\text{i}}^{4} }} - N\frac{{d^{2} v_{\text{i}} }}{{dx_{\text{i}}^{2} }} + kv_{\text{i}} = 0,$$

can be derived; see Table 3. All parameters and variables in Table 3 are clarified in the List of Symbols of this article and the derivation of all expressions is presented in [16].

**Table 3:**
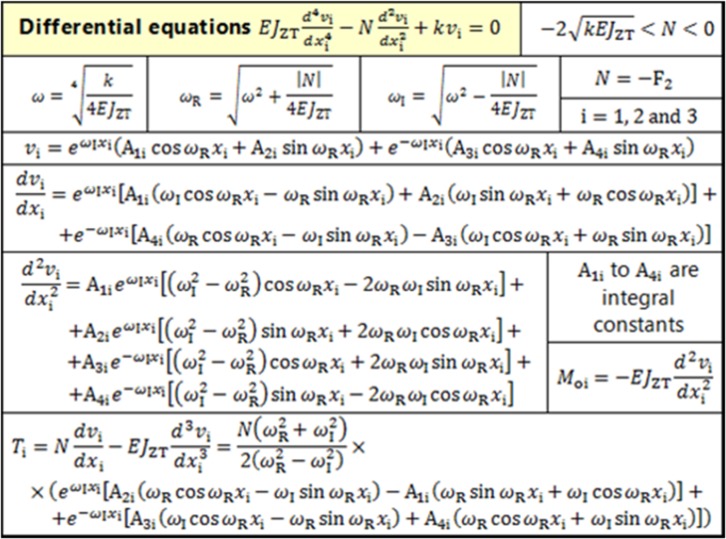
General solutions for a beam rested on an elastic foundation

Note, the beams on elastic foundation often occur in many practical cases for example, solution of building frames and constructions, mining supports etc. too; for example see [16]. However, the applications in the branch of biomechanics are still new.

Let us solve one cancellous screw of length ${\text{L}}$ (i.e. a beam on an elastic foundation) presented in Figs. 9b and 11. The vertical displacement (deflection) $v_{\text{i}} = v\left({x_{\text{i}} } \right)$, for ${\text{i}} = 1;\,2 ;\,3$, must be solved in three sections $x_{\text{i}}$ (i.e. $x_{1} = \left( {0;\,{\text{L}}_{1} } \right)$, $x_{2} = \left( {{\text{L}}_{1} ;\,{\text{L}}_{2} } \right)$ and $x_{3} = \left( {{\text{L}}_{2} ;\,{\text{L}}} \right)$, see Fig. 11 and Table 3, i.e. solution of three differential equations).

Thus, there are twelve constants of integration ${\text{A}}_{{1{\text{i}}}} , \ldots , {\text{A}}_{{4{\text{i}}}}$ which must be solved via twelve boundary conditions at points $x_{1} = 0$  m, $x_{1} = x_{2} = {\text{L}}_{1}$, $x_{2} = x_{3} = {\text{L}}_{2}$ and $x_{3} = {\text{L}}$, i.e.

4 $$M_{{{\text{o}}1}} \left( {x_{1} = 0} \right) = 0 ,\,T_{1} \left( {x_{1} = 0} \right) = 0$$

5 $$\left. {\begin{array}{*{20}c} {v_{1} \left( {x_{1} = {\text{L}}_{1} } \right) - v_{2} \left( {x_{2} = {\text{L}}_{1} } \right) = 0,} \\ {\frac{{dv_{1} }}{{dx_{1} }}\left( {x_{1} = {\text{L}}_{1} } \right) - \frac{{dv_{2} }}{{dx_{2} }}\left( {x_{2} = {\text{L}}_{1} } \right) = 0 ,} \\ {\begin{array}{*{20}c} {M_{o1} \left( {x_{1} = {\text{L}}_{1} } \right) - M_{o2} \left( {x_{2} = {\text{L}}_{1} } \right) = 0,} \\ {T_{1} \left( {x_{1} = {\text{L}}_{1} } \right) - T_{2} \left( {x_{2} = {\text{L}}_{1} } \right) = {\text{F}}_{1} ,} \\ \end{array} } \\ \end{array} } \right\}$$

6 $$\left. {\begin{array}{*{20}c} {v_{2} \left( {x_{2} = {\text{L}}_{2} } \right) - v_{3} \left( {x_{3} = {\text{L}}_{2} } \right) = 0 ,} \\ {\frac{{dv_{2} }}{{dx_{2} }}\left( {x_{2} = {\text{L}}_{2} } \right) - \frac{{dv_{3} }}{{dx_{3} }}\left( {x_{3} = {\text{L}}_{2} } \right) = 0 ,} \\ {\begin{array}{*{20}c} {M_{o2} \left( {x_{2} = {\text{L}}_{2} } \right) - M_{o3} \left( {x_{3} = {\text{L}}_{2} } \right) = 0, } \\ {\begin{array}{*{20}c} { } \\ { T_{2} \left( {x_{2} = {\text{L}}_{2} } \right) - T_{2} \left( {x_{3} = {\text{L}}_{2} } \right) = - {\text{F}}_{1} , } \\ \end{array} } \\ \end{array} } \\ \end{array} } \right\}$$

7 $$M_{o3} \left( {x_{3} = {\text{L}}} \right) = 0 ,\,T_{3} \left( {x_{3} = {\text{L}}} \right) = 0,$$

For example, from the boundary condition Eq. (4) and Table 3 follows $- EJ_{\text{ZT}} \frac{{d^{2} v_{\text{i}} \left( {x_{1} = 0} \right)}}{{dx_{1}^{2} }} = 0$ (i.e. $\frac{{d^{2} v_{\text{i}} \left( {x_{1} = 0} \right)}}{{dx_{1}^{2} }} = 0$). Hence,

$$\begin{aligned} {\text{A}}_{11} e^{0} \left[ {\left( {\omega_{\text{I}}^{2} - \omega_{\text{R}}^{2} } \right)\cos 0 - 2\omega_{\text{R}} \omega_{\text{I}} \sin 0} \right]+ \hfill \\ + {\text{A}}_{21} e^{0} \left[ {\left( {\omega_{\text{I}}^{2} - \omega_{\text{R}}^{2} } \right)\sin 0 + 2\omega_{\text{R}} \omega_{\text{I}} \cos 0} \right]+ \hfill \\ + {\text{A}}_{31} e^{0} \left[ {\left( {\omega_{\text{I}}^{2} - \omega_{\text{R}}^{2} } \right)\cos 0 + 2\omega_{\text{R}} \omega_{\text{I}} \sin 0} \right] +\hfill \\ + {\text{A}}_{41} e^{0} \left[ {\left( {\omega_{\text{I}}^{2} - \omega_{\text{R}}^{2} } \right)\sin 0 - 2\omega_{\text{R}} \omega_{\text{I}} \cos 0} \right] = 0 \hfill \\ \end{aligned}$$

and

8 $$\left. {\begin{array}{*{20}c} {{\text{A}}_{11} \left( {\omega_{\text{I}}^{2} - \omega_{\text{R}}^{2} } \right) + {\text{A}}_{21} 2\omega_{\text{R}} \omega_{\text{I}} + } \\ { + {\text{A}}_{31} \left( {\omega_{\text{I}}^{2} - \omega_{\text{R}}^{2} } \right) - {\text{A}}_{41} 2\omega_{\text{R}} \omega_{\text{I}} = 0. } \\ \end{array} } \right\}$$

Similarly, from the boundary conditions Eqs. (5)–(7), after substitution from Table 3, it is possible to derive a set of twelve linear equations which can be expressed in matrix form as

9 $$\left[ {\mathbf{M}} \right] \times \left\{ {\mathbf{A}} \right\} = \left\{ {\mathbf{B}} \right\} ,$$

where sparse matrix $\left[ {\mathbf{M}} \right]$ with dimension 12 × 12 is defined via submatrices $\left[ {{\mathbf{M}}_{1} } \right]$, $\left[ {{\mathbf{M}}_{2} } \right]$ and $\left[ {{\mathbf{M}}_{3} } \right]$ with dimensions 12 × 4 and column vectors $\left\{ {\mathbf{A}} \right\}$ and $\left\{ {\mathbf{B}} \right\}$ with dimensions 12 × 1 are defined as

10 $$\left[ {\mathbf{M}} \right] = \left[ {\begin{array}{*{20}c} {\left[ {{\mathbf{M}}_{1} } \right]} & {\left[ {{\mathbf{M}}_{2} } \right]} & {\left[ {{\mathbf{M}}_{3} } \right]} \\ \end{array} } \right],$$

11 $$\begin{aligned} \left[ {{\mathbf{M}}_{1} } \right] = \hfill \\ \left[ {\begin{array}{*{20}l} {\omega_{\text{I}} } \hfill & { - \omega_{\text{R }} } \hfill & { - \omega_{\text{I}} } \hfill & { - \omega_{\text{R}} } \hfill \\ s \hfill & t \hfill & s \hfill & { - t} \hfill \\ {af} \hfill & {ah} \hfill & {\frac{f}{a}} \hfill & {\frac{h}{a}} \hfill \\ {a\left( {\omega_{\text{I}} f - \omega_{\text{R}} h} \right)} \hfill & {a\left( {\omega_{\text{R}} f + \omega_{\text{I}} h} \right)} \hfill & {\frac{{ - \omega_{\text{I}} f - \omega_{\text{R}} h}}{ a}} \hfill & {\frac{{\omega_{\text{R}} f - \omega_{\text{I}} h}}{a}} \hfill \\ {a\left( {fs - th} \right)} \hfill & {a\left( {hs + tf} \right)} \hfill & {\frac{fs + th}{ a}} \hfill & {\frac{hs - tf}{a}} \hfill \\ { - a\left( {\omega_{\text{I}} f + \omega_{\text{R}} h} \right)} \hfill & {a\left( {\omega_{\text{R}} f - \omega_{\text{I}} h} \right)} \hfill & {\frac{{\omega_{\text{I}} f - \omega_{\text{R}} h}}{ a}} \hfill & {\frac{{\omega_{\text{R}} f + \omega_{\text{I}} h}}{a}} \hfill \\ 0 \hfill & 0 \hfill & 0 \hfill & 0 \hfill \\ \vdots \hfill & \vdots \hfill & \vdots \hfill & \vdots \hfill \\ 0 \hfill & 0 \hfill & 0 \hfill & 0 \hfill \\ \end{array} } \right] \hfill \\ \end{aligned}$$

12 $${\left[ {{\mathbf{M}}_{2} } \right]\,} = { \left[ {\begin{array}{*{20}l} 0  & 0  & 0  & 0  \\0  & 0  & 0  & 0  \\ -af  & -ah  & -f/a  & -h/a  \\ {a(\omega_{\text{R}}h-\omega_{\text{I}}f)}  & {-a(\omega_{\text{R}}f+\omega_{\text{I}}h)}  & { \frac{\omega_{\text{I}}f+\omega_{\text{R}}h}{ a}}  & {\frac{ \omega_{\text{I}}h-\omega_{\text{R}}f}{a}}  \\ {a(th-fs)}  & {-a(hs+tf)}  & {\frac{-fs-th}{a}}  & {\frac{tf-hs}{a}}  \\ {a(\omega_{\text{I}}f+\omega_{\text{R}}h)}  & {a(\omega_{\text{I}}h-\omega_{\text{R}}f)}   & {\frac{\omega_{\text{R}}h-\omega_{\text{I}}f}{a}}   & {\frac{-\omega_{\text{R}}f-\omega_{\text{I}}h}{a}}   \\ {bj}   & {bq}   & {j/b}   & {q/b}   \\ {b(\omega_{\text{I}}j-\omega_{\text{R}}q)}   & {b(\omega_{\text{R}}j+\omega_{\text{I}}q)}   & {\frac{-\omega_{\text{I}}j-\omega_{\text{R}}q}{b}}   & {\frac{\omega_{\text{R}}j-\omega_{\text{I}}q}{b}}   \\ b(sj-tq)   & b(sq+tj)   & {\frac{sj+tq}{b}}   & {\frac{sq-tj}{b}}   \\ {-b\left( {\omega_{\text{R}}q+\omega_{\text{I}}j} \right)}   & {b\left( {\omega_{\text{R}}j-\omega_{\text{I}}q} \right)}   & {\frac{\omega_{\text{I}}j-\omega_{\text{R}}q}{b}}   & {\frac{\omega_{\text{R}}j+\omega_{\text{I}}q}{b}}   \\ 0   & 0   & 0   & 0   \\ 0   & 0   & 0   & 0     \\ \end{array} } \right]} $$

13 $$_{{\left[ {\begin{array}{*{20}c} 0 & 0 & 0 & 0 \\ \vdots & \vdots & \vdots & \vdots \\ 0 & 0 & 0 & 0 \\ {-bj} & {-bq} & -j/b & -q/b \\ {b(\omega_{\text{R}} q - \omega_{\text{I}} j)} & { - b(\omega_{\text{R}} j + \omega_{\text{I}} q)} & {\frac{{\omega_{\text{I}} j + \omega_{\text{R}} q}}{b}} & {\frac{{\omega_{\text{I}} q - \omega_{\text{R}} j}}{b}} \\ {b(tq - sj)} & { - b(sq + tj)} & {\frac{ - sj - tq}{b}} & {\frac{tj - sq}{b}} \\ {b(\omega_{\text{R}} q + \omega_{\text{I}} j)} & {b(\omega_{\text{I}} q - \omega_{\text{R}} j)} & {\frac{{\omega_{\text{R}} q - \omega_{\text{I}} j}}{b}} & {\frac{{ - \omega_{\text{R}} j - \omega_{\text{I}} q}}{b}} \\ {c(sp - tr)} & {c(sr + tp)} & {\frac{sp + tr}{c}} & {\frac{sr - tp}{c}} \\ { - c(\omega_{\text{R}} r + \omega_{\text{I}} p)} & {c(\omega_{\text{R}} p - \omega_{\text{I}} r)} & {\frac{{\omega_{\text{I}} p - \omega_{\text{R}} r}}{c}} & {\frac{{\omega_{\text{R}} p + \omega_{\text{I}} r}}{c}} \\ \end{array} } \right]}}^{{\left[ {{\mathbf{M}}_{3} } \right] = }}$$

14 $$\left\{ {\mathbf{A}} \right\} = \left\{ {\begin{array}{*{20}c} {{\text{A}}_{11} } \\ {{\text{A}}_{21} } \\ {\begin{array}{*{20}c} {{\text{A}}_{31} } \\ {{\text{A}}_{41} } \\ {\begin{array}{*{20}c} {{\text{A}}_{12} } \\ {{\text{A}}_{22} } \\ {\begin{array}{*{20}c} {{\text{A}}_{32} } \\ {{\text{A}}_{42} } \\ {\begin{array}{*{20}c} {{\text{A}}_{13} } \\ {{\text{A}}_{23} } \\ {\begin{array}{*{20}c} {{\text{A}}_{33} } \\ {{\text{A}}_{43} } \\ \end{array} } \\ \end{array} } \\ \end{array} } \\ \end{array} } \\ \end{array} } \\ \end{array} } \right\} ,$$

15 $$\varvec{ }\left\{ {\mathbf{B}} \right\} = \frac{{2{\text{F}}_{1} s}}{{{\text{F}}_{2} \left( {\omega_{\text{R}}^{2} + \omega_{\text{I}}^{2} } \right)}} \times \,\varvec{ }\left[ {\begin{array}{*{20}c} 0 & 0 & 0 & 0 & 0 & 1 & 0 & 0 & 0 & - 1 & 0 & 0 \\ \end{array} } \right]^{{\mathbf{T}}} ,$$

where parameters

16 $$\left. {\begin{array}{*{20}c} {a = e^{{\omega_{{\text{I}}} {\text{L}}_{1} }} , b = e^{{\omega_{{\text{I}}} {\text{L}}_{2} }} , c = e^{{\omega_{{\text{I}}} {\text{L}}}} ,} \\ {f = \cos \left( {\omega_{{\text{R}}} {\text{L}}_{1} } \right), h = \sin \left( {\omega_{{\text{R}}} {\text{L}}_{1} } \right),} \\ {\begin{array}{*{20}c} { j = \cos \left( {\omega_{{\text{R}}} {\text{L}}_{2} } \right), p = \cos \left( {\omega_{{\text{R}}} {\text{L}}} \right), } \\ {\begin{array}{*{20}c} {q = \sin \left( {\omega_{{\text{R}}} {\text{L}}_{2} } \right), r = \sin \left( {\omega_{{\text{R}}} {\text{L}}} \right) ,} \\ {s = \omega_{{\text{I}}}^{2} - \omega_{{\text{R}}}^{2} , t = 2\omega_{R} \omega_{\text{I}} } \\ \end{array} } \\ \end{array} } \\ \end{array} } \right\}$$

Note, the derived Eq. (8) is written in the 2nd row in the Eq. (11).

This analytical approach is easy to solve. It leads to the solution of twelve linear equations. As a further step, the application of nonlinearities in elastic foundations is also possible, for example see [16, 23, 25, 35], i.e. the application of the Central Finite Difference Method or the Finite Element Method in connection with the iterative Newton Method.

## Numerical Model and its Solution and Evaluation

By the solution of a set of linear Eq. (9), i.e.

17 $$\left\{ {\mathbf{A}} \right\} = \left[ {\mathbf{M}} \right]^{ - 1} \times \left\{ {\mathbf{B}} \right\},$$

the constants of integration ${\text{A}}_{{1{\text{i}}}} , \ldots , {\text{A}}_{{4{\text{i}}}}$ can be found; the general results are shown in Table 3.

Hence, displacements, slopes, bending moments $M_{o}$, shearing forces $T$ and normal forces $N$ can be evaluated over the whole length of the cancellous screw (beam).

In mechanics, $N$, $T$ are internal forces and $M_{o}$ is internal moment. These induce mechanical stresses in bodies. Stresses are important for the reliability assessment of bodies.

**Fig. 14:**
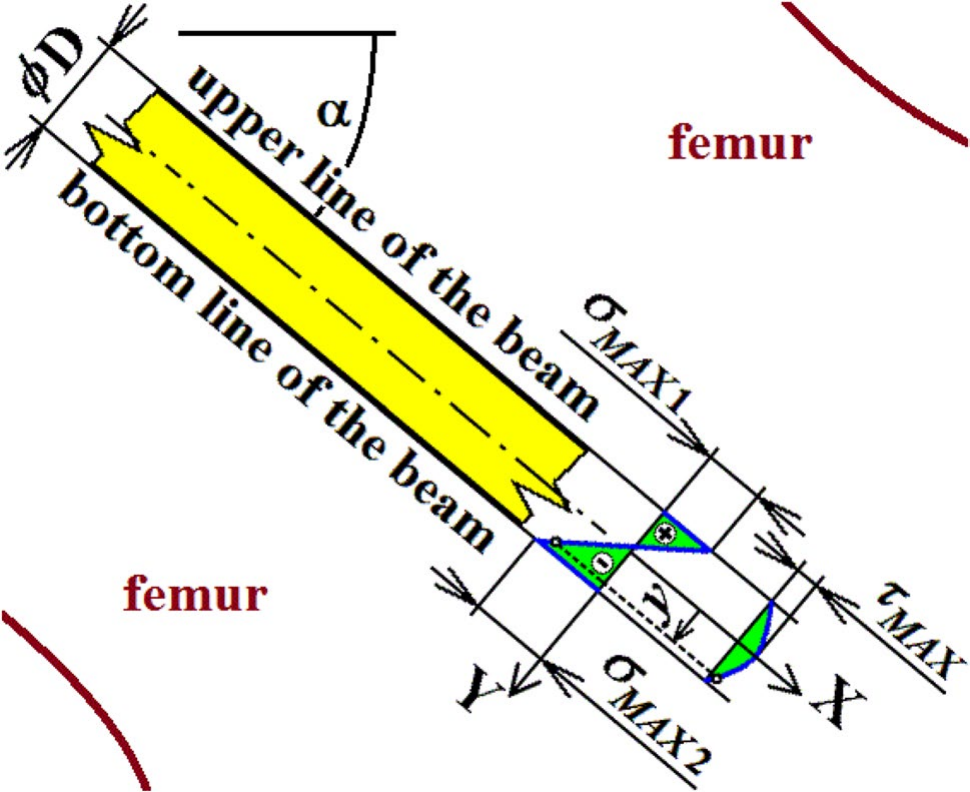
Stress evaluation in the cancellous screw (beam, full cross-section)

**Fig. 15:**
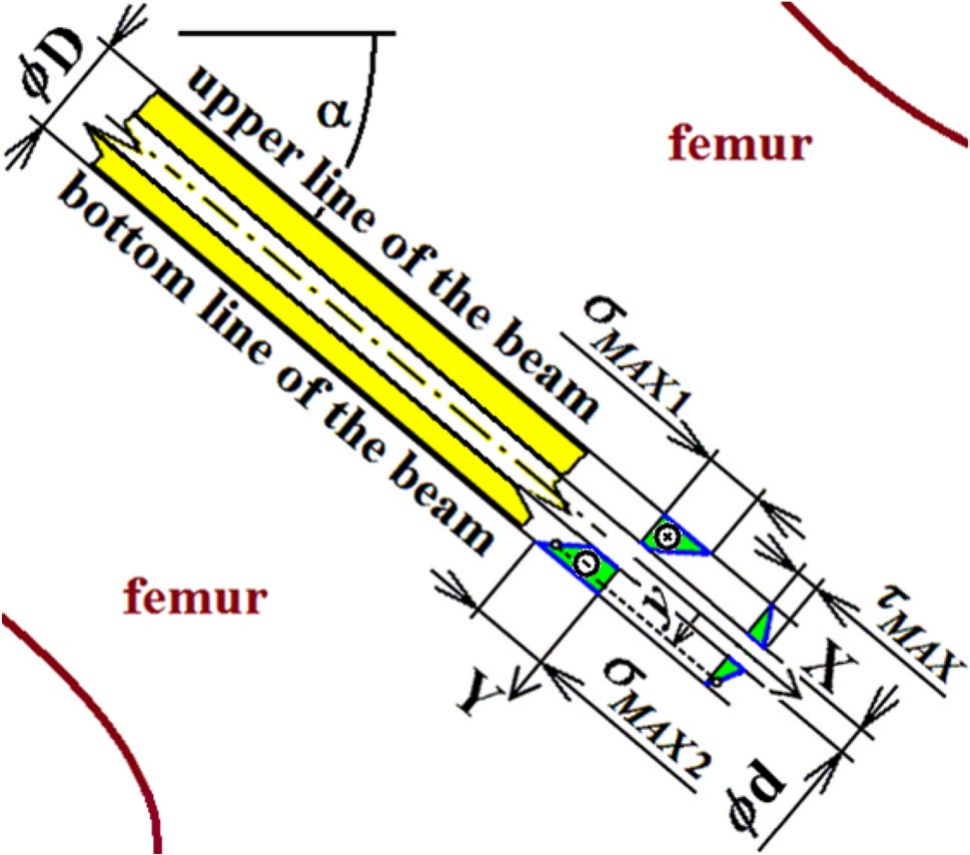
Stress evaluation in the cancellous screw (beam, cannulated cross-section)

Because the normal stresses are constant over the whole length of the screw, maximal stresses (i.e. the influence of bending moments and normal forces) are prescribed by the expression

18 $$\sigma_{MAX1} = \frac{N}{A} - \frac{{M_{oMAX} }}{{W_{o} }} ,\sigma_{MAX2} = \frac{N}{A} + \frac{{M_{oMAX} }}{{W_{o} }},$$

see Figs. 14 and 15. Parameter $A$ is the cross-sectional area of a beam and $W_{o}$ is the section modulus of a beam in bending; see List of Symbols.

Maximal shear stress is prescribed by the expression

19 $$\tau_{MAX} = \frac{{4T_{MAX} }}{3A},$$

for a full cross-section; see Fig. 14; and

20 $$\tau_{MAX} = \frac{{2T_{MAX} }}{A},$$

for a cannulated cross-section of a cancellous screw; see Fig. 15.

Safety factor is a term describing the structural capacity of a system beyond its expected loads or actual loads. Essentially it expresses how much stronger the system is than it usually needs to be for an intended load. Our definition of safety factor ${\text{S}}_{{{\text{R}}_{\text{e}} }}$ is a ratio of Yield strength ${\text{R}}_{\text{e}}$ (i.e. material parameter) to the absolute value of maximal (bending + compression) stress $\left| {\sigma_{\text{MAX}} } \right|$ (i.e. load response parameter)

21 $$S_{{{\text{R}}_{\text{e}} }} = \frac{{{\text{R}}_{\text{e}} }}{{\left| {\sigma_{MAX} } \right|}},$$

see Fig. 16, and

22 $$\left| {\sigma_{MAX} } \right| = { \hbox{max} }\left( {\left| {\sigma_{MAX1} } \right|, \left| {\sigma_{MAX2} } \right|} \right)$$

**Fig. 16:**
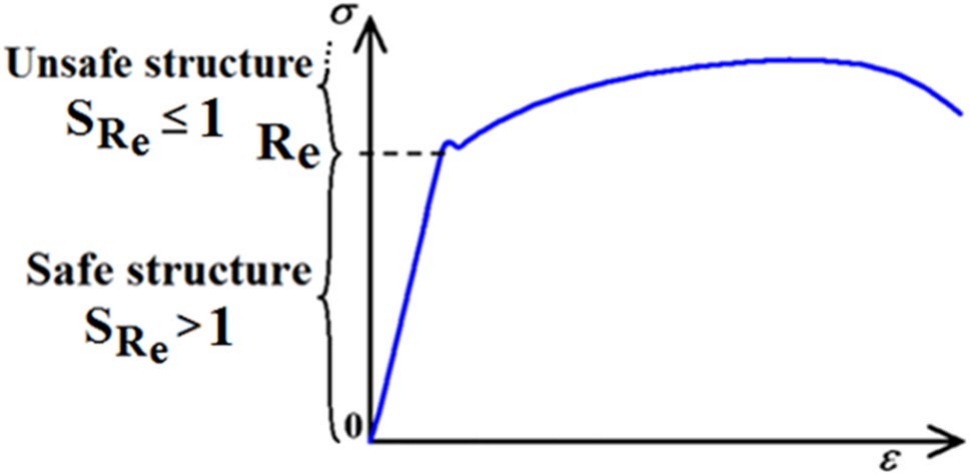
Stress–strain diagram of material—definition of safe and unsafe structure

**Fig. 17:**
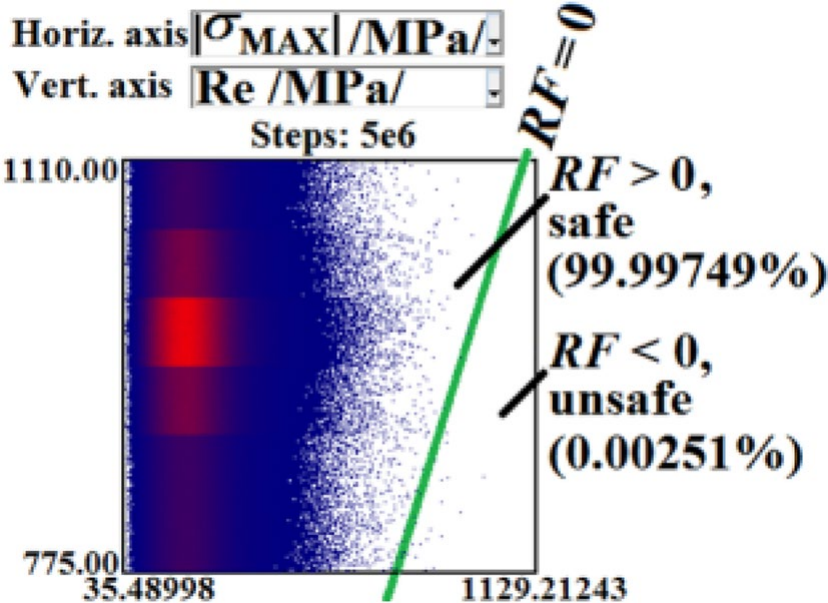
2D histogram of reliability function *RF* (result of 5 × 10^6^ Monte Carlo random simulations); see [18]—mentioned in this article but not presented in full here

In general, ${\text{S}}_{{{\text{R}}_{\text{e}} }}$ is of stochastic quality. In this article (i.e. the first part of our solution), the stochastic approach is not applied. However, in the future continuation of this work, the stochastic approach can be applied via the Simulation-Based Reliability Assessment (SBRA) Method (i.e. Monte Carlo approach); see references [3, 4, 16–18, 35, 36] and Fig. 17.

## Results

### Deterministic Results - Cancellous Screw with Full Cross-Section Made up from Stainless Steel (*α* = 50 deg, L = 0.09 m)

The solution of three cancellous screws with full cross-section (shank diameter D = 4.5 mm) made of stainless steel, see Eq. (17), Table 1, Figs. 8, 10, 11 and 14, is performed for the input parameters prescribed in Table 4.

**Table 4:**
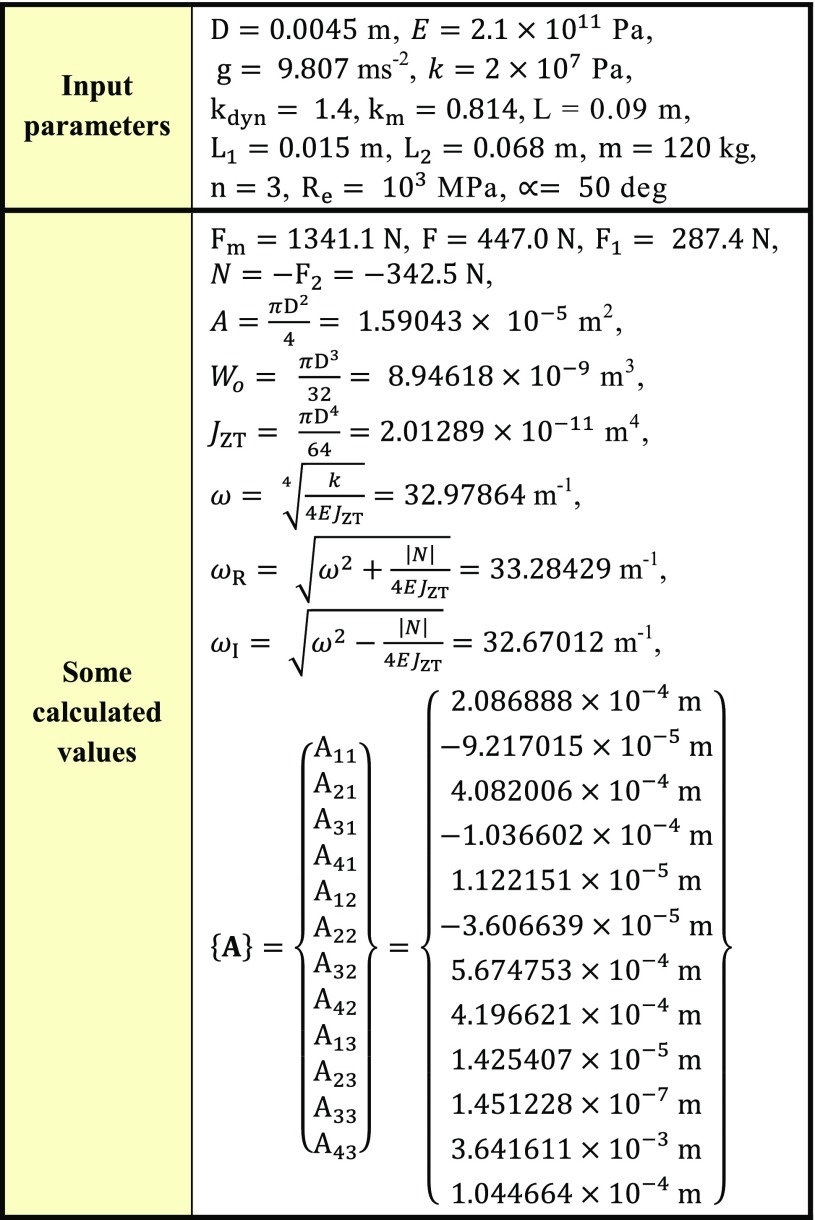
Input parameters for a cancellous screw with full cross-section made up from stainless steel

**Fig. 18:**
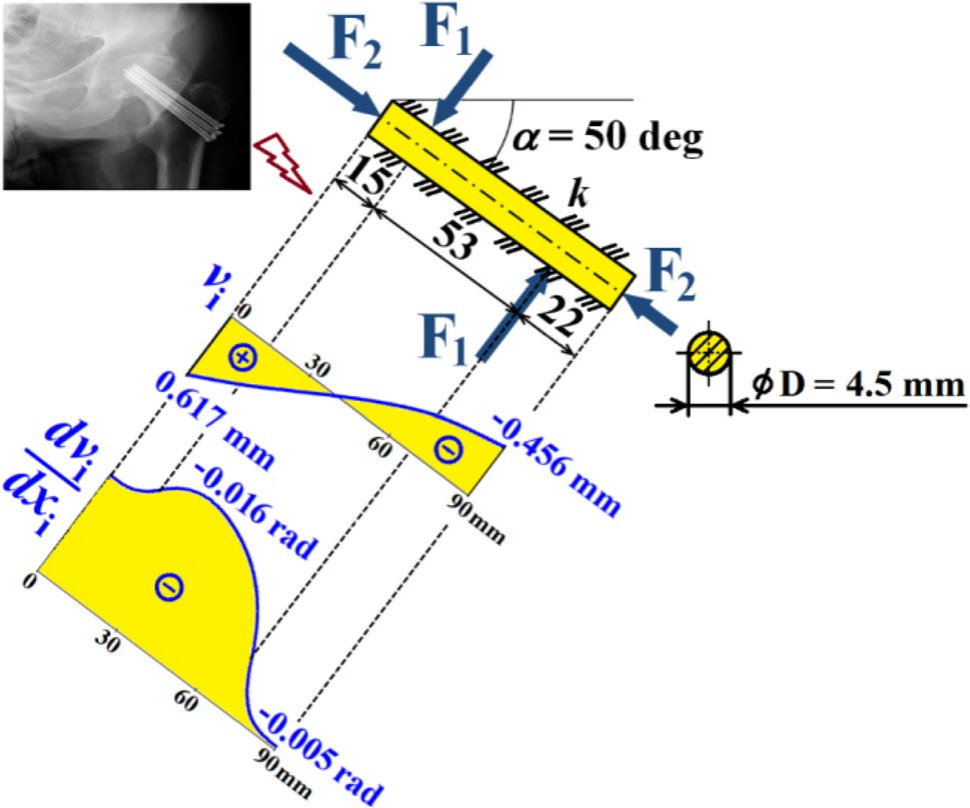
Dependencies of displacement $v_{\text{i}}$ and slope $\frac{{dv_{\text{i}} }}{{dx_{\text{i}} }}$ in one cancellous screw (full cross-section, stainless steel)

**Fig. 19:**
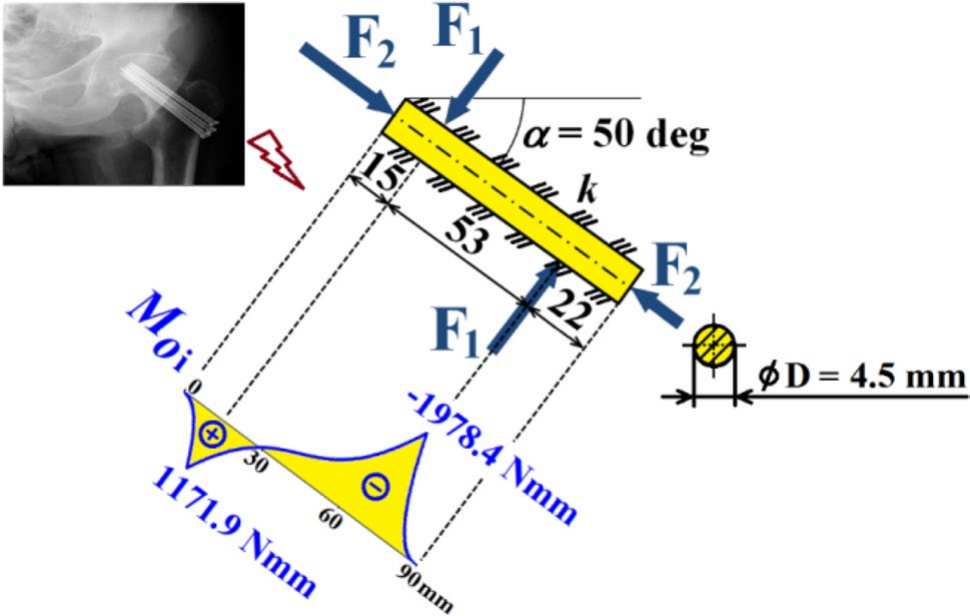
Dependence of bending moment $M_{{o{\text{i}}}}$ in one cancellous screw (full cross-section, stainless steel)

**Fig. 20:**
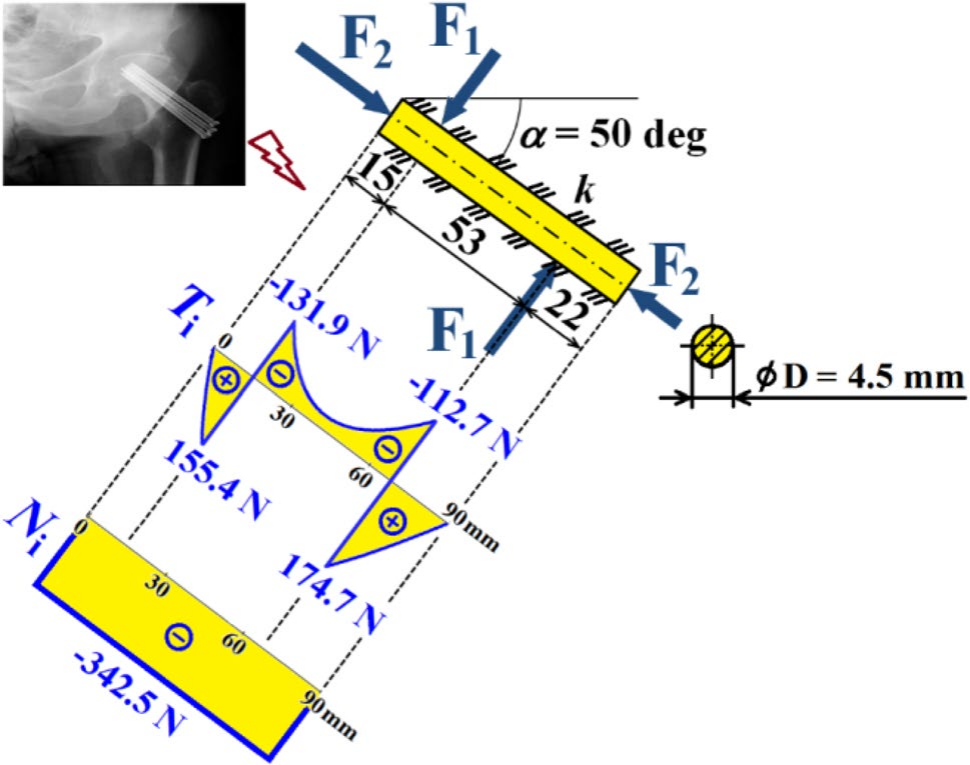
Dependencies of shearing force $T_{\text{i}}$ and normal force $N$ in one cancellous screw (full cross-section, stainless steel)

Hence dependencies of $v_{\text{i}}$, $\frac{{dv_{\text{i}} }}{{dx_{\text{i}} }}$, $M_{{o{\text{i}}}}$, $T_{\text{i}}$ and $N$ can be calculated; see diagrams in Figs. 18, 19 and 20.

From the presented results, the maximal values for vertical displacement $v_{\text{MAX}}$, shearing forces $T_{\text{MAX}}$, and bending moments $M_{{o{\text{MAX}}}}$ can be evaluated. Finally, stresses $\sigma_{MAX1}$, $\sigma_{MAX2}$, $\tau_{MAX}$ and safety factor $S_{{{\text{R}}_{\text{e}} }}$, see Figs. 14, 18, 19 and 20 Eqs. (18), (20), (21) and (22), can be evaluated; see Table 5.

**Table 5:** Some important output parameters for a cancellous screw with full cross-section made up from stainless steel

Some output values	$$\begin{aligned} v_{MAX} & = 0.617{\text{mm}},\,T_{MAX} = 174.66{\text{N}}, \\ \tau_{MAX} & = 14.64{\text{MPa,}}\,M_{{{\text{o}}MAX}} = - 1978.44{\text{Nmm,}} \\ \sigma_{MAX1} & = 199.62{\text{MPa,}}\,\sigma_{MAX} = \sigma_{MAX2} = - 242.68{\text{MPa,}} \\ {\mathbf{SR}}_{{\mathbf{e}}} & {\mathbf{ = 4}}{\mathbf{.12}} \\ \end{aligned}$$

The main results are discussed in the Discussion and Conclusions.

### Deterministic Results- Cancellous Screw with Cannulated Cross-Section Made up from Ti6Al4V Material (*α* = 50 deg, L = 0.09 m)

The solution of three cancellous screws with cannulated cross-section (shank diameter D = 5 mm, cannulation diameter d = 1.8 mm) made of Ti6Al4V material, see Eq. (17), Table 1, Figs. 8, 10, 11 and 15, is performed for the input parameters prescribed in Table 6.

**Table 6:**
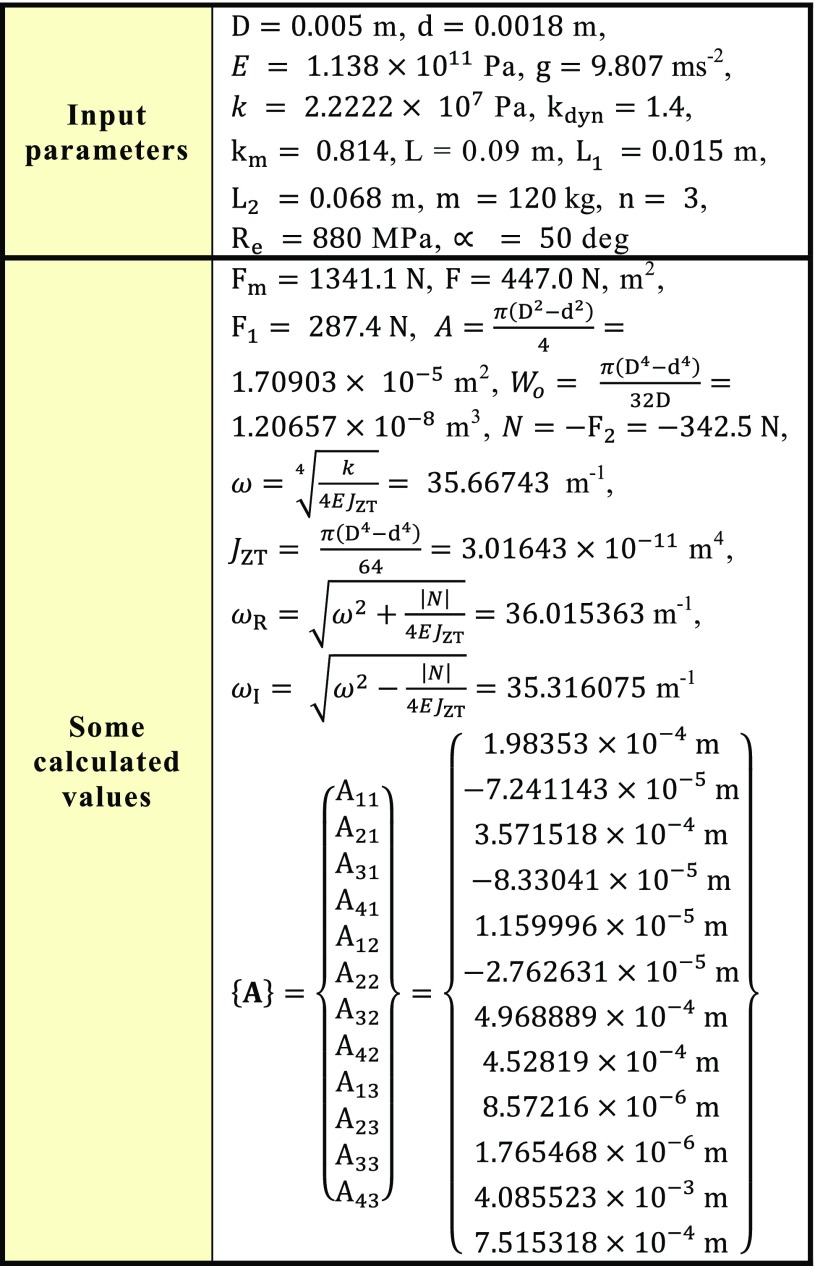
Input parameters for a cancellous screw with cannulated cross-section made up from Ti6Al4V material

**Fig. 21:**
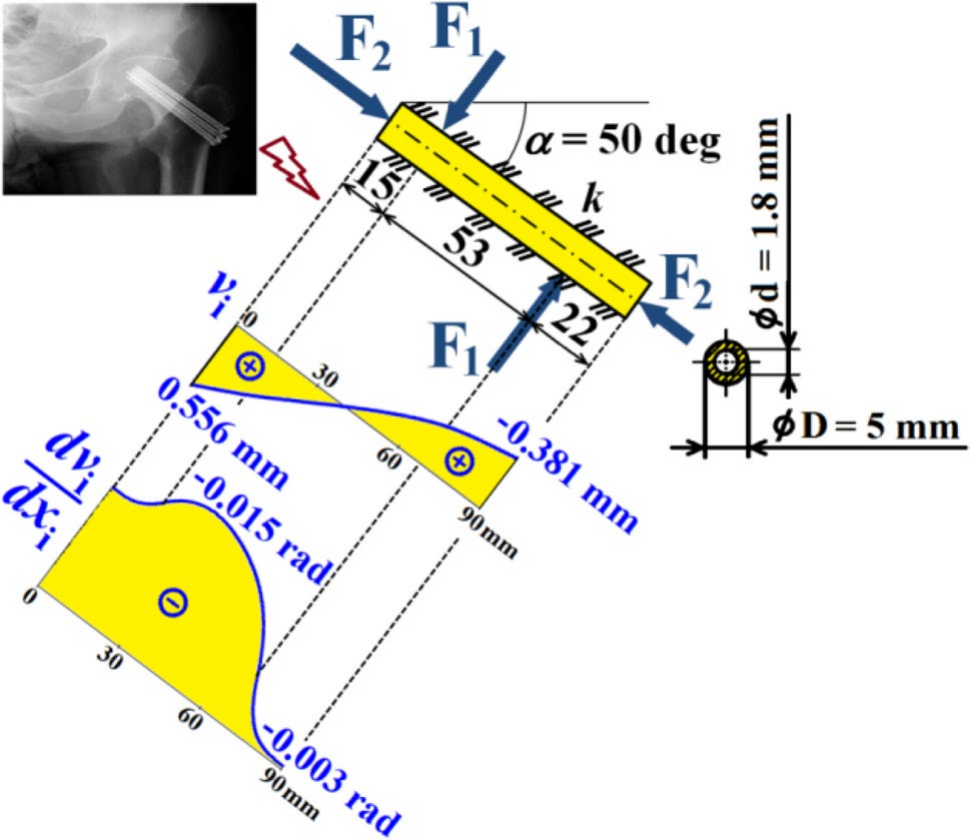
Dependencies of displacement $v_{\text{i}}$ and slope $\frac{{dv_{\text{i}} }}{{dx_{\text{i}} }}$ in one cancellous screw (cannulated cross-section, Ti6Al4V material)

**Fig. 22:**
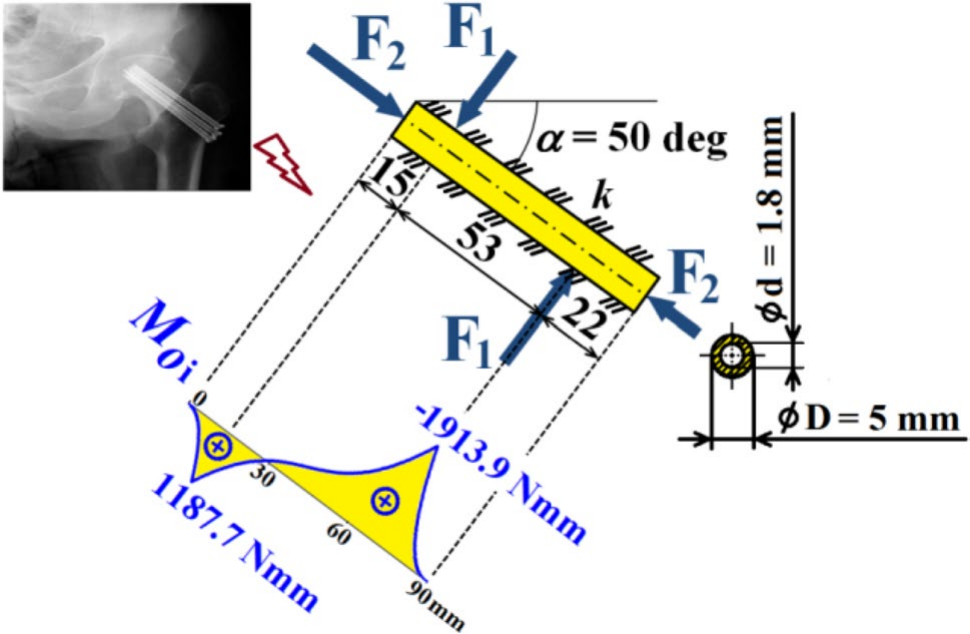
Dependence of bending moment $M_{{o{\text{i}}}}$ in one cancellous screw (cannulated cross-section, Ti6Al4V material)

**Fig. 23:**
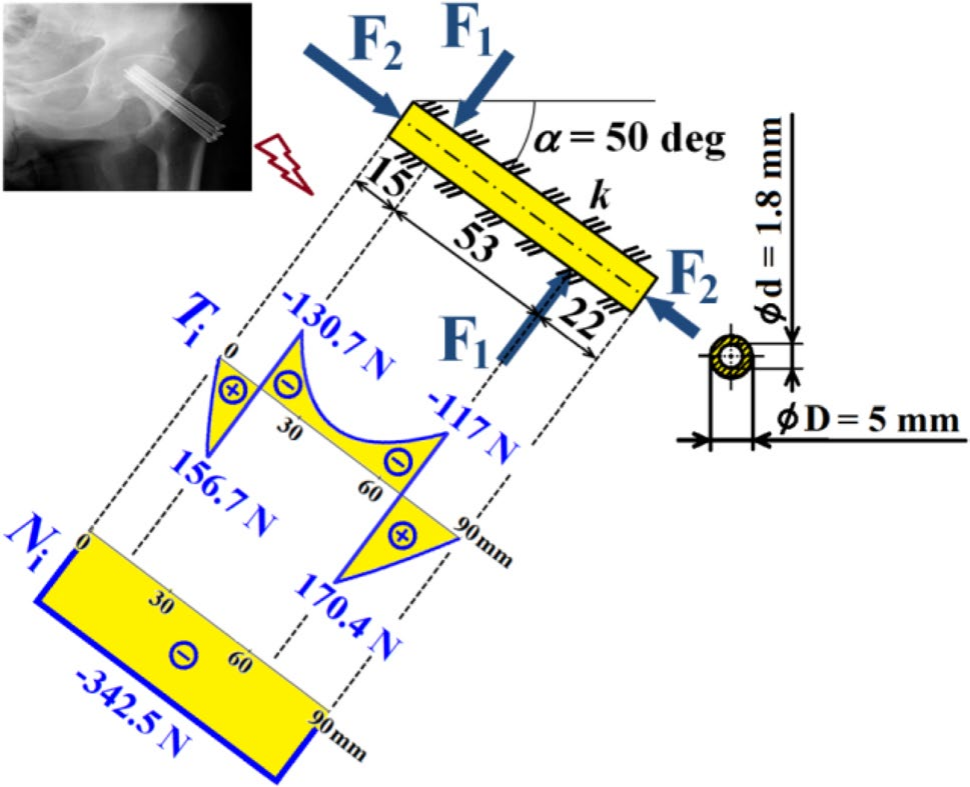
Dependencies of shearing force $T_{\text{i}}$ and normal force $N$ in one cancellous screw (cannulated cross-section, Ti6Al4V material)

Hence, dependencies of $v_{\text{i}}$, $\frac{{dv_{\text{i}} }}{{dx_{\text{i}} }}$, $M_{{o{\text{i}}}}$, $T_{\text{i}}$ and $N$ can be calculated; see Figs. 21, 22 and 23.

From the presented results, the maximal values for vertical displacement $v_{\text{MAX}}$, shearing forces $T_{\text{MAX}}$, and bending moments $M_{{o{\text{MAX}}}}$ can be evaluated. Finally, stresses $\sigma_{MAX1}$, $\sigma_{MAX2}$, $\tau_{MAX}$ and safety factor $S_{{{\text{R}}_{\text{e}} }}$, see Fig. 15 and Eqs. (18), (20), (21) and (22), can be evaluated; see Table 7.

**Table 7:** Some important output parameters for a cancellous screw with cannulated cross-section made up from Ti6Al4V material

Some output values	$$\begin{aligned} v_{MAX} & = 0.556\text{ mm, } T_{MAX} = 170.35{\text{ N,}} \\ \tau_{MAX} & = 19.94\text{ MPa, } M_{{{\text{o}}MAX}} = - 1913.96{\text{ Nmm}},\, \\ \sigma_{MAX1} & = 138.59\,MPa,\,\, \\ \sigma_{MAX} & = \sigma_{MAX2} = - 176.66\text{ MPa, }\varvec{S}_{{{\mathbf{R}}_{{\mathbf{e}}} }} = {\mathbf{4.98}} \\ \end{aligned}$$

The main results are discussed in the Discussion and Conclusions.

## Discussion

Proximal femoral neck “collum femoris” fractures remain a vexing clinical problem in traumatology and are one of the most common types of trauma. One possible treatment method for femoral neck fractures is the application of cancellous screws (i.e. lag spongious screws) made of Ti6Al4V or stainless steel material.

This paper therefore aims to present both a basic medical perspective (i.e. types and methods of treatment and possible complications/problems) and an engineering perspective (i.e. our original and simple numerical model for strength analyses and its evaluation) for cancellous screws (i.e. for one possible method of treatment).

The presented analytical model of cancellous screws is based on the theory of beams on an elastic (Winkler’s) foundation, where the bone is approximated by the elastic foundation (an acceptable and suitable simplification of the complicated reality of mechanical contact and interaction between the cancellous screw and bone tissue).

Three screws of length 90 mm were applied in parallel positions on the elastic foundation (i.e. applied in femur bone). The value for quasi-dynamic forces (acting in one screw) were derived according to the parameters of the patient.

According to the 2nd order theory and the theory of beams on an elastic foundation, a set of three 4th order linear differential equations is introduced together with 12 boundary conditions. Matrix notation is used for expressing the acquisition of constants of integration.

The solution (i.e. examples of two calculations) is performed for cancellous screws with full cross-section or cannulated cross-section made of stainless steel or Ti6Al4V material. Displacement, slopes, bending moments, normal forces, shearing forces and normal stresses are calculated and presented in diagrams. Maximal shear stresses and total maximal stresses are calculated and evaluated.

Finally, the safety factor (i.e. the ratio of yield limit to maximal stress) is determined for the given type of cancellous screw. The values of the safety factor for two examples are found 4.12 (cancellous screw with full cross-section made of stainless steel) and 4.98 (cancellous screw with cannulated cross-section made of Ti6Al4V material). *Therefore the application of cancellous screws in the treatment of “collum femoris” fractures is suitable, safe and recommended (i.e. orthopaedists and traumatologists can use it for the treatment of patients).*

**Fig. 24:**
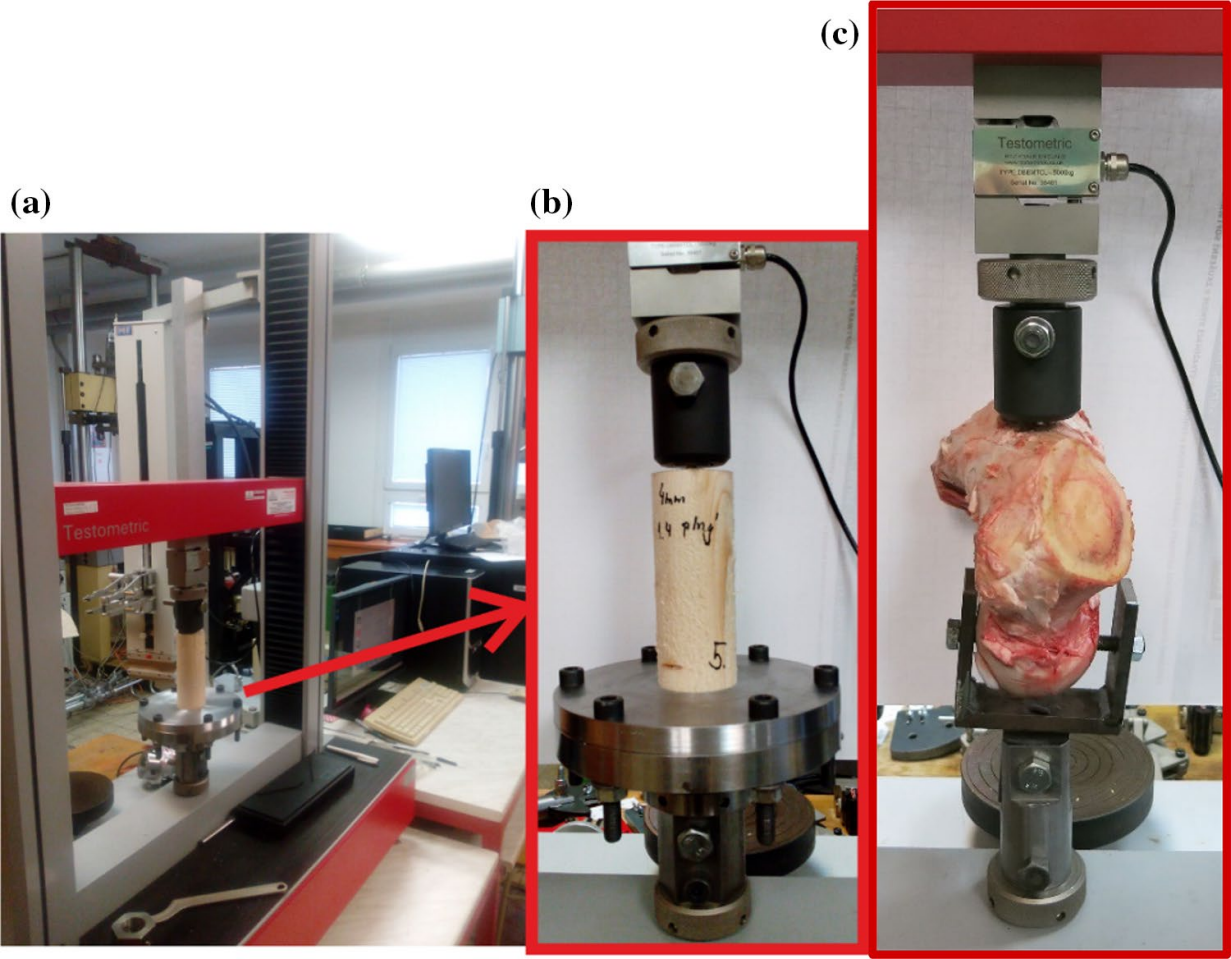
Experiments **a** testing machine, **b** cancellous screw in spruce wood, **c** cancellous screw in bovine femur (mentioned in this article but not presented in full here)

The derivation and rapid solutions of our own simple numerical model open up a new avenue for further applications using a stochastic approach (i.e. millions of solutions with random inputs and outputs can be easily simulated and evaluated). The Simulation-Based Reliability Assessment (SBRA) Method (i.e. the direct Monte Carlo approach etc.) can be applied. This method can respect the real variability of inputs and outputs via truncated histograms. The application of the SBRA Method is a new and modern trend in mechanics/biomechanics. Therefore, the application of the SBRA method connected with the probabilistic reliability assessment and laboratory experiments of cancellous screws is the main focus of the next part of this article (i.e. future continuation) of this work; see for example [3, 4, 16–18, 35, 36] and Figs. 17 and 24.

Figure 24 shows the screw being pulled out of spruce wood and a bovine femur (i.e. initial experiments in a study of force dependencies and the behaviour of bone as an elastic foundation). These experiments represent preparations for more demanding cadaver tests.

As a future extension of our work, see [18, 23, 25], the elastic foundation can also be approximated via nonlinear functions. However, this leads to the solution of three nonlinear 4^th^ order differential equations. This solution can apply the Central Difference Method with the iterative Newton Method; e.g. see work in [23, 25]. This also offers a good and desirable improvement.

**Fig. 25:**
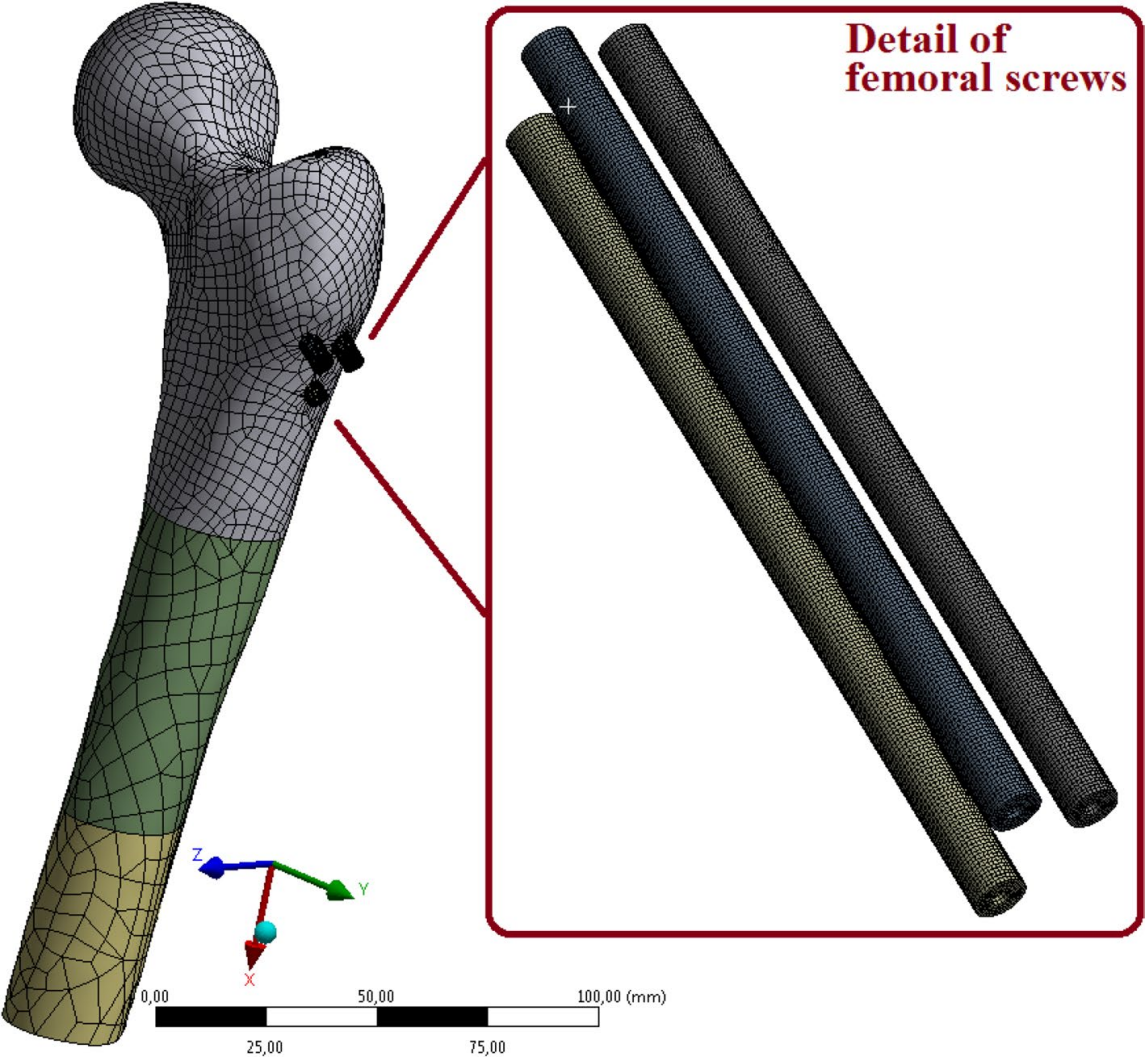
Simple 3D FE model of three cannulated cancellous screws in the femur for verification of the presented results (mentioned in this article but not presented in full here)

**Fig. 26:**
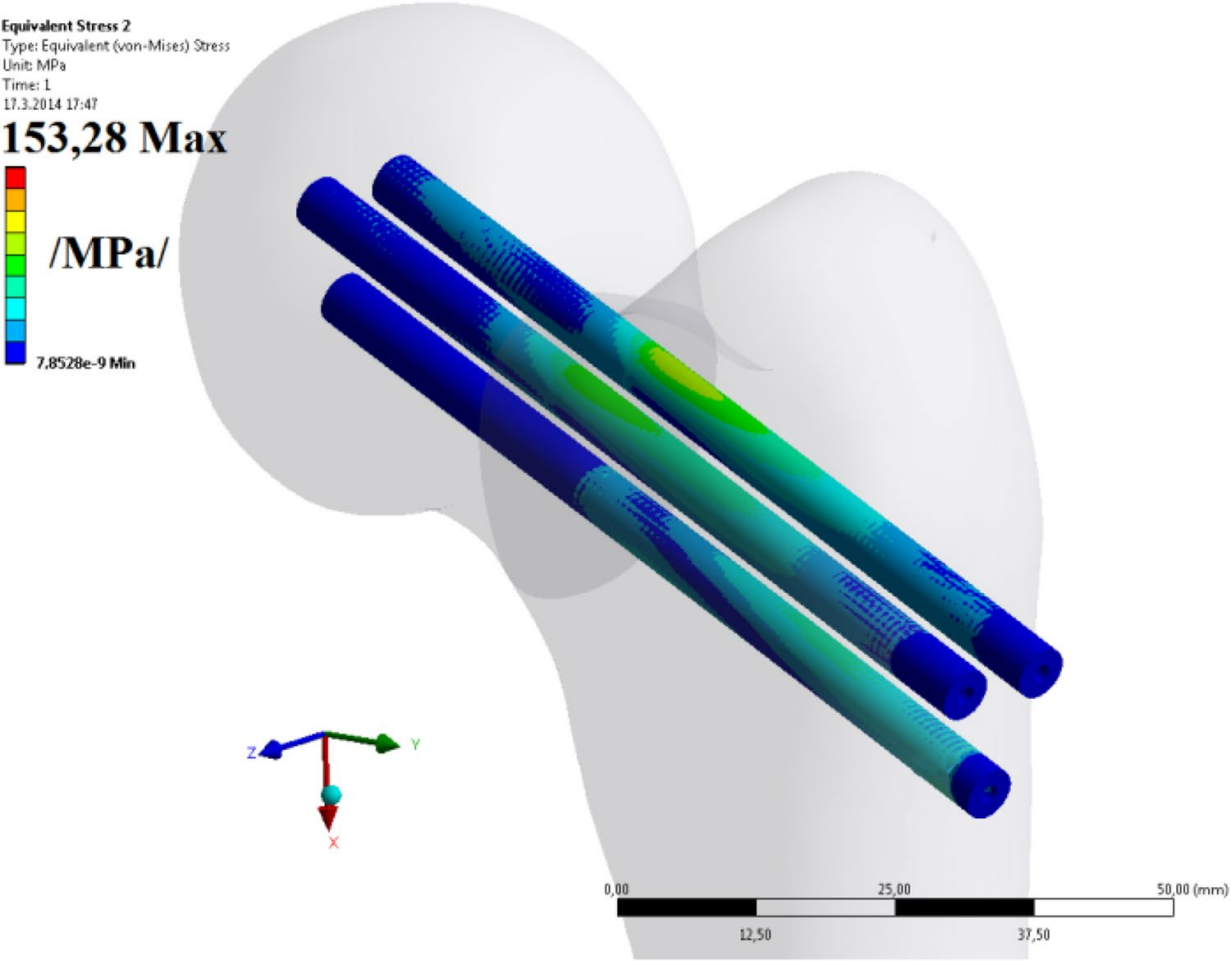
Simple 3D FE solution of three cannulated cancellous screws in the femur for verification of the presented results (mentioned in (mentioned in this article but not presented in full here)

The presented results (i.e. displacements and stresses) were compared (tested) with a simple 3D FE model (though not in this article) with adequate results; see Figs. 25 and 26. The relative differences between the analytical and FE models for maximal stresses, strains and displacements are ≤ 6.6%, which is sufficient. For more information see [18].

However, obtaining the results by FEM (ANSYS software) takes a much longer time than when using our original 2D beam solution as presented in this article. The mentioned application of 3D FE model will be published in future; see [18] and Figs. 25 and 26.

On the other hand, our model can also be used for calculating/assessing inappropriate or unacceptable positions of cancellous screws (changes of angles $\propto$, length ${\text{L}}$, number of screws, parallel or nonparallel positions of screws, screws can or cannot be in contact with the femoral neck cortex, etc.).

## Conclusions

The article discusses a basic medical perspective on collum femoris fractures with the focus on their treatment via cancellous (i.e. femoral) screws.

The simple planar model of a cancellous screw in a femur as a beam on an elastic foundation is applied. 2^nd^ order theory is applied, and materials, dimensions, loading, differential equations etc. and their solutions are described. A biomechanical evaluation (i.e. evaluation of deformations and stresses) is carried out. The computational model as a whole is characterized by its quick solution and high variability of possible screw insertion positions.

According to the results (see Table 5 and 7), the safety factor ranges from 3 to 5 (i.e. 300% to 500% safety that undesirable plastic deformation will not occur). The cancellous screws are safe, and they are recommended as suitable for treatment of collum femoris fractures.

Other possibilities for future research and developments are mentioned and discussed.

*Hence, this article has presented new methods and ideas and demonstrated their applications in biomechanics, centred around a new, simple approach to the solution of cancellous screws with applications in the branch of traumatology and orthopaedics.*

## List of Symbols

*A*: Cross-sectional area of cancellous (femoral) screw /m^2^/
A_li,…,_A_4i,_{**A**}: Integral constants and vector of integral constants (output variables) /m/
$a$, $b$, $c$: Parameters of matrix $\left[ {\mathbf{M}} \right]$ /1/
$\left\{ {\mathbf{B}} \right\}$: Vector of left side (input variables) /1/
${\text{D}}$,${\text{d}}$: Outer (shank) and inner (cannulation) diameter of cancellous screw (input variables) /m/
*E*: Elastic modulus of cancellous screw (input variable) /Pa/
*e*: Euler’s number (i.e. base of the natural logarithm) /1/
F: Quasi-dynamic force acting in one cancellous screw (input variable) /N/
F_m_: Total loading quasi-dynamic force acting in caput femoris /N/
F_1_, F_2_: Tangential and axial force acting in one cancellous screw /N/
$f$: Parameter of matrix $\left[ {\mathbf{M}} \right]$ /1/
${\text{g}}$: Gravitational acceleration (input variable) /ms^−2^/
$h$: Parameter of matrix $\left[ {\mathbf{M}} \right]$ /1/
${\text{i}}$: Index of section of cancellous screw (i.e. ${\text{i}} = 1, 2 {\text{ and }} 3$)
$J_{\text{ZT}}$: Principal quadratic moment of cross-sectional area of cancellous screw /m^4^/
$j$: Parameter of matrix $\left[ {\mathbf{M}} \right]$ /1/
$k$: Elastic foundation stiffness (i.e. bone stiffness, input variable) /Nm^−2^/
${\text{k}}_{\text{dyn}} , {\text{k}}_{\text{m}}$: Dynamic force and mass reduction coefficients /1/
${\text{L}},{\text{L}}_{1}$, ${\text{L}}_{2}$: Total length and local lengths of cancellous screws (input variables) /m/
$\left[ {\mathbf{M}} \right],\varvec{ }\left[ {{\mathbf{M}}_{1} } \right]$, $\left[ {{\mathbf{M}}_{2} } \right]$, $\varvec{ }\left[ {{\mathbf{M}}_{3} } \right]$: Matrix of equations and its submatrices (input variables) /1/
$M_{\text{oi}}$, $M_{\text{oMAX}}$: Bending moments in sections of screw and maximal bending moment in screw in absolute value (output variables) /Nm/
$N$: Normal force in cancellous screw /N/
${\text{m}}$: Entire mass of a patient (input variable) /kg/
$$m$$: Distributed moment /N/
${\text{n}}$: Coefficient of inequality in the division of force ${\text{F}}$ /1/
$p$: Parameter of matrix $\left[ {\mathbf{M}} \right]$ /1/
q: distributed loading /Nm^−1^/
$q$: Parameter of matrix $\left[ {\mathbf{M}} \right]$ /1/
${\text{q}}_{\text{R}}$: Reaction force in the elastic foundation /Nm^−1^/
$r$: Parameter of matrix $\left[ {\mathbf{M}} \right]$ /1/
$r_{\text{i}}$: Radius of curvature/m/
${\text{R}}_{\text{e}}$: Yield stress of material of cancellous screw /MPa/
$RF$: Reliability function (output variable) /MPa/
$s$: Parameter of matrix $\left[ {\mathbf{M}} \right]$ /m^−2^/
$S_{{{\text{R}}_{\text{e}} }}$: Safety factor of cancellous screw (otput variable) /1/
$t$: Parameter of matrix $\left[ {\mathbf{M}} \right]$ /m^−2^/
$T_{\text{i}}$, $T_{MAX}$: Shearing forces in sections of cancellous screw and maximal shearing force in absolute value (otput variables) /N/
${\text{t}}_{1}$, ${\text{t}}_{2}$: Temperatures in the upper and bottom line of beam/^o^C/or/K/
$v_{1}$, $v_{2}$, $v_{3}$, $v_{\text{i}}$, $v_{MAX}$: Deflection (i.e. vertical displacement) in sections of cancellous screw and its maximum (output variables) /m/
$\frac{{dv_{\text{i}} }}{{dx_{\text{i}} }}$: Slope of a screw (beam, output variable)/rad/
$W_{\text{o}}$: Section modulus in bending /m^3^/
$x_{1}$, $x_{2}$, $x_{3}$, $x_{\text{i}}$: Cartesian coordinates in sections /m/
$y$: Cartesian coordinate in sections /m/
$\propto$: Cancellous screw angle (input variables) /deg/
$\varepsilon$: Strain (output variable) /1/
$\sigma$: Stress (output variable) /MPa/
$\sigma_{MAX}$, $\sigma_{MAX1}$, $\sigma_{MAX2}$: Global maximal normal stress in cancellous screw and local maximal normal stresses in cancellous screw (in absolute values, output variables) /MPa/
$\tau_{MAX}$: Maximal shear stress in cancellous screw (in absolute values, output variable) /MPa/
$\omega$, $\omega_{\text{R}}$, $\omega_{\text{I}}$: Parameters of the numerical solutions /m^−1^/
